# Minor intron splicing revisited: identification of new minor intron-containing genes and tissue-dependent retention and alternative splicing of minor introns

**DOI:** 10.1186/s12864-019-6046-x

**Published:** 2019-08-30

**Authors:** Anouk M. Olthof, Katery C. Hyatt, Rahul N. Kanadia

**Affiliations:** 1 Physiology and Neurobiology Department, University of Connecticut, Storrs, CT 06269 USA; 2 Institute of Systems Genomics, University of Connecticut, Storrs, CT 06269 USA

**Keywords:** MIDB, Minor spliceosome, Alternative splicing, Tissue-specificity, Minor intron retention

## Abstract

**Background:**

Mutations in minor spliceosome components such as U12 snRNA (cerebellar ataxia) and U4atac snRNA (microcephalic osteodysplastic primordial dwarfism type 1 (MOPD1)) result in tissue-specific symptoms. Given that the minor spliceosome is ubiquitously expressed, we hypothesized that these restricted phenotypes might be caused by the tissue-specific regulation of the minor spliceosome targets, i.e. minor intron-containing genes (MIGs). The current model of inefficient splicing is thought to apply to the regulation of the ~ 500 MIGs identified in the U12DB. However this database was created more than 10 years ago. Therefore, we first wanted to revisit the classification of minor introns in light of the most recent reference genome. We then sought to address specificity of MIG expression, minor intron retention, and alternative splicing (AS) across mouse and human tissues.

**Results:**

We employed position-weight matrices to obtain a comprehensive updated list of minor introns, consisting of 722 mouse and 770 human minor introns. These can be found in the Minor Intron DataBase (MIDB). Besides identification of 99% of the minor introns found in the U12DB, we also discovered ~ 150 new MIGs. We then analyzed the RNAseq data from eleven different mouse tissues, which revealed tissue-specific MIG expression and minor intron retention. Additionally, many minor introns were efficiently spliced compared to their flanking major introns. Finally, we identified several novel AS events across minor introns in both mouse and human, which were also tissue-dependent. Bioinformatics analysis revealed that several of the AS events could result in the production of novel tissue-specific proteins. Moreover, like the major introns, we found that these AS events were more prevalent in long minor introns, while retention was favoured in shorter introns.

**Conclusion:**

Here we show that minor intron splicing and AS across minor introns is a highly organised process that might be regulated in coordination with the major spliceosome in a tissue-specific manner. We have provided a framework to further study the impact of the minor spliceosome and the regulation of MIG expression. These findings may shed light on the mechanism underlying tissue-specific phenotypes in diseases associated with minor spliceosome inactivation. MIDB can be accessed at https://midb.pnb.uconn.edu.

**Electronic supplementary material:**

The online version of this article (10.1186/s12864-019-6046-x) contains supplementary material, which is available to authorized users.

## Background

Splicing of introns is required for the expression of most eukaryotic genes. A subset of mammalian genes contains both major and minor introns, therefore requiring both the major (U1, U2, U4, U6 and U5) and the minor (U11, U12, U4atac, U6atac and U5) spliceosome, respectively [1]. In these minor intron-containing genes (MIGs), the majority of introns are usually spliced by the major spliceosome, with only one or two introns being spliced by the minor spliceosome. Regardless, mis-splicing of these few minor introns can have detrimental consequences, as highlighted by diseases caused by mutation in minor spliceosome components. For example, mutation in *RNU4ATAC*, which encodes for U4atac snRNA, has been linked to microcephalic osteodysplastic primordial dwarfism type I (MOPD1), Roifman syndrome and Lowry-Wood syndrome [2–5]. Since U4atac snRNA is ubiquitously expressed, mutation in this gene is expected to result in systemic inhibition of the minor spliceosome. Consequently, the expectation is that all tissues of the developing embryo would be affected. Instead, the cardinal symptoms that characterize these diseases include microcephaly and skeletal dysplasia, suggesting that the developing brain and bone are more prone to the loss of minor splicing than other tissues [2, 3]. One way to explain this paradox of systemic minor spliceosome inhibition resulting in tissue-specific phenotypes would be that the MIGs are expressed, spliced and/or alternatively spliced in a tissue-specific manner. As a consequence, certain tissues might be more susceptible to loss of minor spliceosome function. Indeed, there is evidence that cell-types are differentially affected by loss of minor spliceosome. For instance, we recently reported a mouse model for microcephaly, in which we inactivated the minor spliceosome through ablation of U11 snRNA in the dorsal telencephalon [6]. We found that loss of minor spliceosome function affected symmetrically dividing radial glial cells more severely than intermediate progenitor cells and neurons, underscoring cell type-specific susceptibility to loss of minor splicing. Moreover, RNAseq revealed that expression of MIGs was generally not affected, despite elevated retention of many minor introns [6]. On the other hand, splicing of several minor introns was not affected in the U11-null dorsal telencephalon [6]. This dynamic response to loss of minor splicing in a cell-type specific manner suggests that minor intron splicing and MIG expression might also be highly regulated in WT tissues. Therefore, we here wanted to determine whether minor intron splicing and MIG expression showed tissue-specific regulation.

## Results

### Identification of new minor introns in the mouse and human genome

**Fig. 1:**
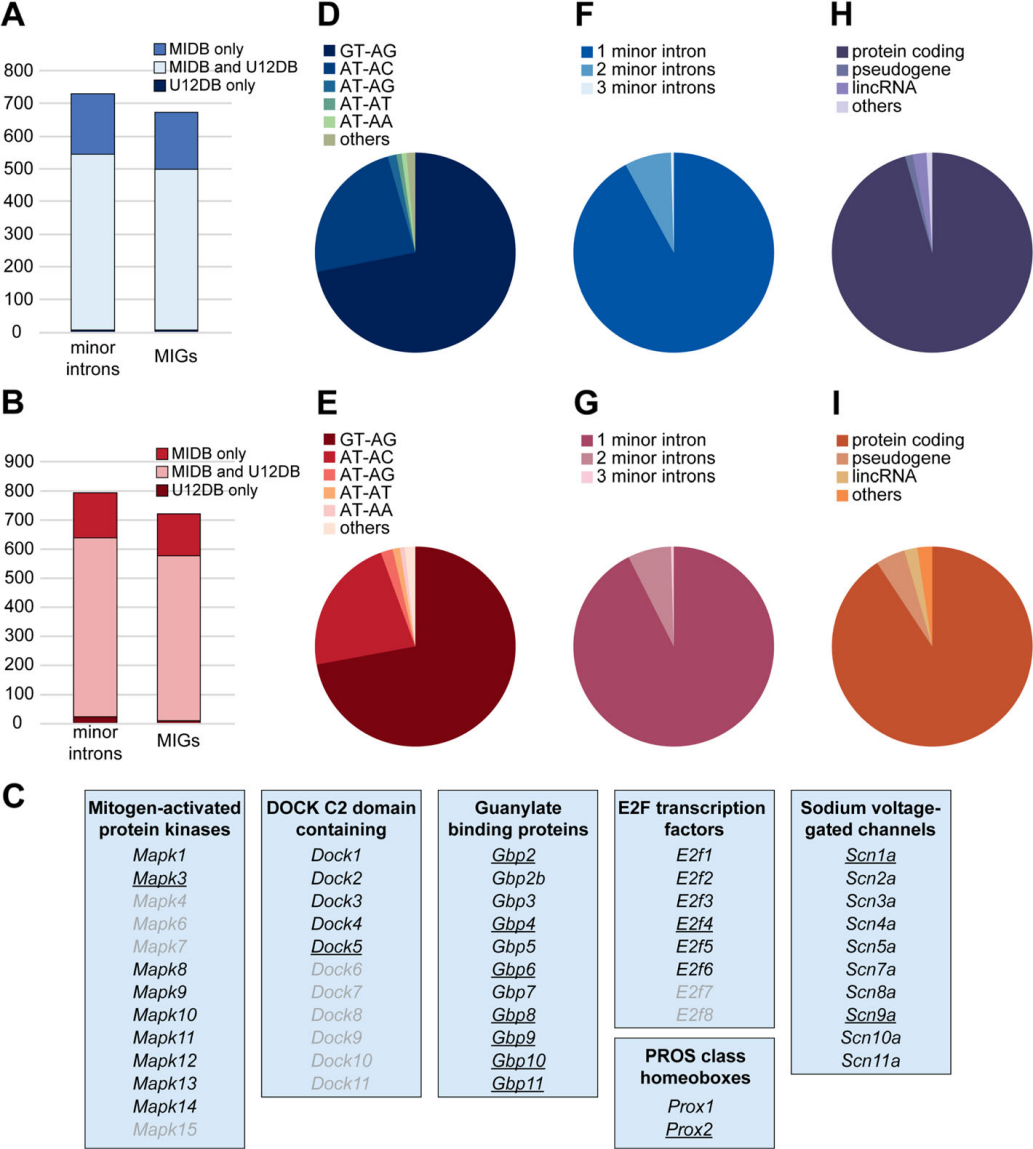
Identification of new minor introns in the mouse and human genome. **a**-**b** Stacked bargraph showing the number of minor introns and MIGs identified in the mouse (**a**) and human (**b**) genome by the U12DB and MIDB. **c** Overview of mouse gene families that contain MIGs (black text) and were newly identified in MIDB (underlined). **d-e** Piechart showing the distribution of mouse (**d**) and human (**e**) minor introns based on their terminal dinucleotides. **f-g** Piechart showing the distribution of mouse (**f**) and human (**g**) MIGs with one, two or three minor introns. **h-i** Piechart showing the gene types of all mouse (**h**) and human (**i**) MIGs

Historically, minor introns were defined based on their divergent terminal dinucleotides (AT-AC) [7]. However, since the initial discovery of minor introns, several AT-AC introns were shown to be spliced by the major spliceosome [8, 9]. As such, not only the terminal dinucleotides, but the entire 5′ and 3′ splice site, as well as the branch point sequence are now taken into account in order to define minor introns. In 2006, a comprehensive analysis of all annotated introns was performed by two independent groups [10, 11]. By employing position-weight matrices (PWM) of the consensus sequences of all annotated introns, they were able to classify introns as major or minor. However, these studies are over 10 years old, and the genome annotation has been significantly updated since. Thus, we first wanted to obtain an updated comprehensive list of all minor introns in the mouse and human genome (Ensembl assembly 95). Using PWMs, we identified 722 minor introns in canonical mouse nascent transcripts and 770 minor introns in canonical human nascent transcripts. (Fig. 1a-b; Additional file 9: Table S1). Of the mouse minor introns, 184 (25%) were not previously identified by the U12DB, whereas 7 (1%) minor introns were identified by the U12DB, but not found in our analysis (Fig. 1a). These 722 mouse minor introns were distributed across 666 genes, of which 172 MIGs were not previously reported in the U12DB (Fig. 1a). For humans, we newly identified 153 (20%) minor introns, while 23 (3%) minor introns from the U12DB were not classified as such in our analysis (Fig. 1b). The 770 identified minor introns were found in 714 MIGs, of which 145 were not previously reported in the U12DB (Fig. 1b). Several of the newly identified minor introns were part of gene families that already had members identified as MIGs. For instance, we now identified a minor intron in *E2f4*, which is part of the E2F family that already had *E2f1, E2f2, E2f3, E2f5* and *E2f6* listed as MIGs in the U12DB (Fig. 1c). Similarly, our analysis identified new minor introns in other gene families known to contain several MIGs, such as the mitogen-activated protein kinases, guanylate binding proteins, DOCKs, and voltage-gated sodium channels (Fig. 1c).

Next, we investigated the terminal dinucleotides of all the identified minor introns, and found that ~ 72% of the minor introns were of the GT-AG type, whereas only ~ 24% and ~ 22% was of the AT-AC type in mouse and human, respectively (Fig. 1d-e). The distribution of minor introns across MIGs was similar between mouse and human in that for both genomes there were 50 MIGs that contained 2 minor introns, and 3 MIGs that contained 3 minor introns, while the remaining MIGs only contained a single minor intron (Fig. 1f-g). Following this analysis, we wanted to interrogate the type of genes in which these minor introns were found. Interestingly, we found that while the majority of MIG transcripts were protein-coding, 8 MIGs in the mouse genome were predicted to be pseudogenes (Fig. 1h). Moreover, 15 MIGs were long intergenic non-coding RNAs (lincRNAs) (Fig. 1h). Similarly, in the human genome, 34 MIGs were predicted to be pseudogenes, and 15 MIGs lincRNAs (Fig. 1i). In all, we have identified ~ 150 new MIGs compared to previous minor intron annotations (Fig. 1a-b) [10, 11].

Finally, we wanted to compare the identity of MIGs between mouse and human. To this end, we curated the orthologs listed in the Ensembl and NCBI database, and found that 586 genes contained a minor intron in both mouse and human genome (Additional file 10: Table S2) [10]. As such, there were 80 mouse-specific MIGs and 128 human-specific MIGs (Additional file 10: Table S2).

### MIGs show dynamic expression across mouse and human tissues

**Fig. 2:**
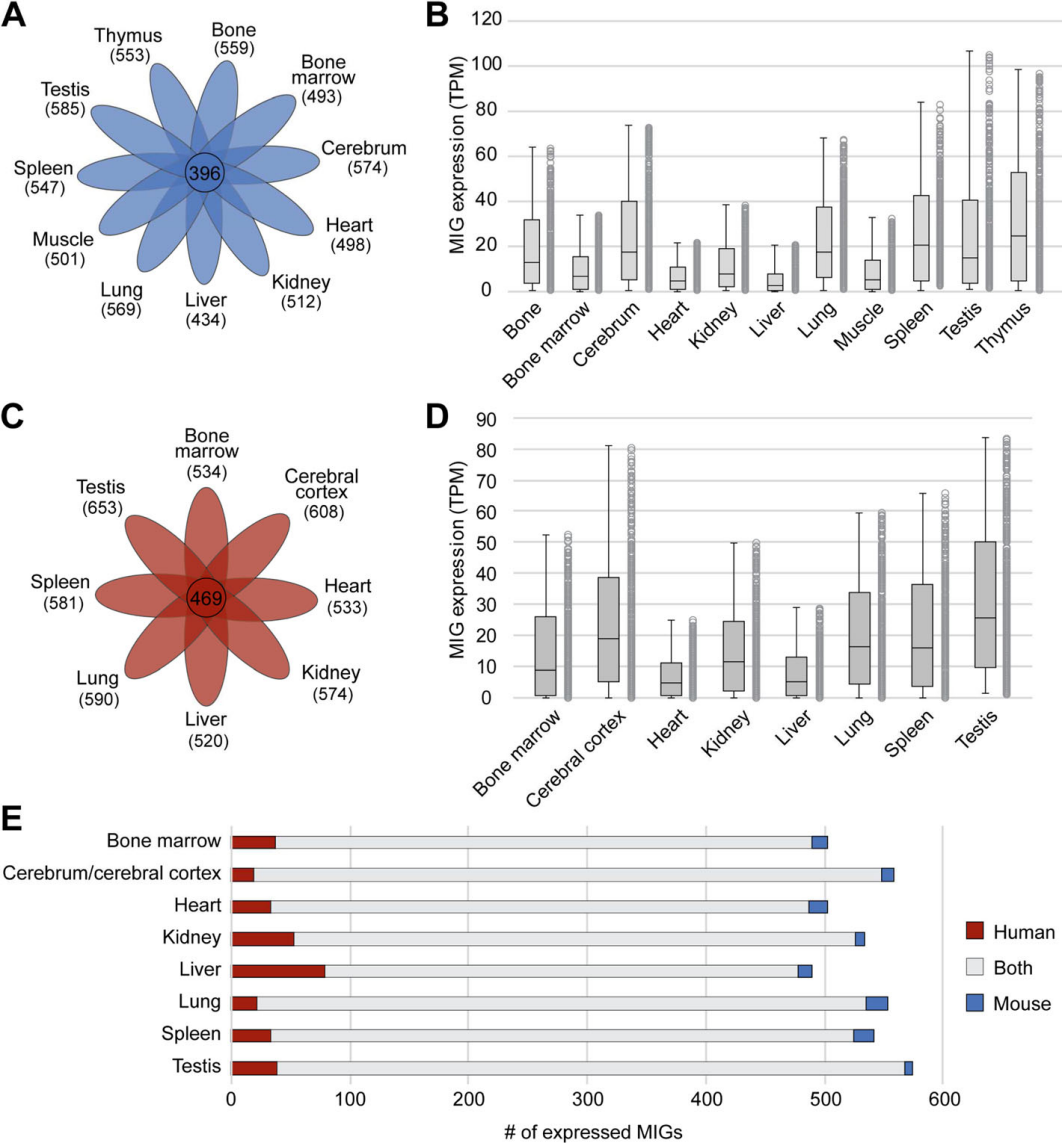
Expression of minor intron-containing genes is dynamic across mouse and human tissues. **a** Venn diagram showing the overlap of MIGs expressed > 1 TPM in eleven mouse tissues. **b** Boxplots representing the 10th–90th percentile of MIG expression in TPM across mouse tissues. **c** Venn diagram showing the overlap of MIGs expressed > 1 TPM in eight human tissues. **d** Boxplots representing the 10th–90th percentile of MIG expression in TPM across human tissues. **e** Stacked bargraph showing the number of MIGs expressed (> 1 TPM) in each tissue in mouse (blue), human (red), or both (grey). TPM = transcripts per million

With our updated list of minor introns, we next wanted to characterize the expression of MIGs across tissues. To this end, we interrogated RNAseq data from eleven different adult WT mouse tissues and eight different tissues from healthy human adults [12, 13]. By setting the expression threshold at 1 TPM, we found that the fewest MIGs were expressed in the mouse liver (434 out of 666; 65%), whereas the most MIGs were expressed in the testis (585 out of 666; 88%) (Fig. 2a). Furthermore, there were 396 MIGs (59%) expressed in all of the eleven mouse tissues (Fig. 2a). Next, we wanted to determine whether tissues with a high number of expressed MIGs, such as the cerebrum and testis, also expressed these MIGs at a high level. Indeed, we found that not only many MIGs were expressed in the testis and cerebrum, but they were also expressed at relatively high levels (Fig. 2b; Additional file 11: Table S3). Moreover, we found that MIGs were expressed at high levels in the mouse spleen and thymus (Fig. 2b). In contrast, heart and liver, the two tissues that had the least number of MIGs expressed, also had low levels of MIG expression (Fig. 2b; Additional file 11: Table S3). Thus, it is not only the number of expressed MIGs, but also the level at which these MIGs are expressed that differs between tissues. Importantly, this finding is conserved in humans, where we analyzed MIG expression in eight different tissues (Fig. 2c). Like in mouse, we found that MIGs were expressed at the lowest levels in the human heart and liver, and at the highest levels in the cerebral cortex and testis (Fig. 2d; Additional file 11: Table S3).

Next, we wanted to determine whether the specific MIGs that were expressed in each tissue were the same in mouse and human. For this, we used the 586 MIGs that were conserved across both genomes to interrogate MIG expression across eight tissues. Overall, we found that if a MIG was expressed in a mouse tissue, it was also expressed in that same tissue in human (Fig. 2e). Only few MIGs showed differences in expression between mouse and human tissues. For example, we found 18 MIGs that were expressed in the mouse lung, but not in the human lung. Similarly, there were 22 MIGs expressed in the human lung, but not in mouse. Regardless, the majority (512) of MIGs were expressed in both the mouse and human lung (Fig. 2e). We observed a similar trend in most other tissues, except in the liver, where there was a relatively high number of MIGs (79) exclusively expressed in humans (Fig. 2e). In all, we found that MIG expression is mostly conserved between mouse and human and shows dynamic changes across tissues.

### Tissue-enriched MIGs participate in specific biological functions

**Fig. 3:**
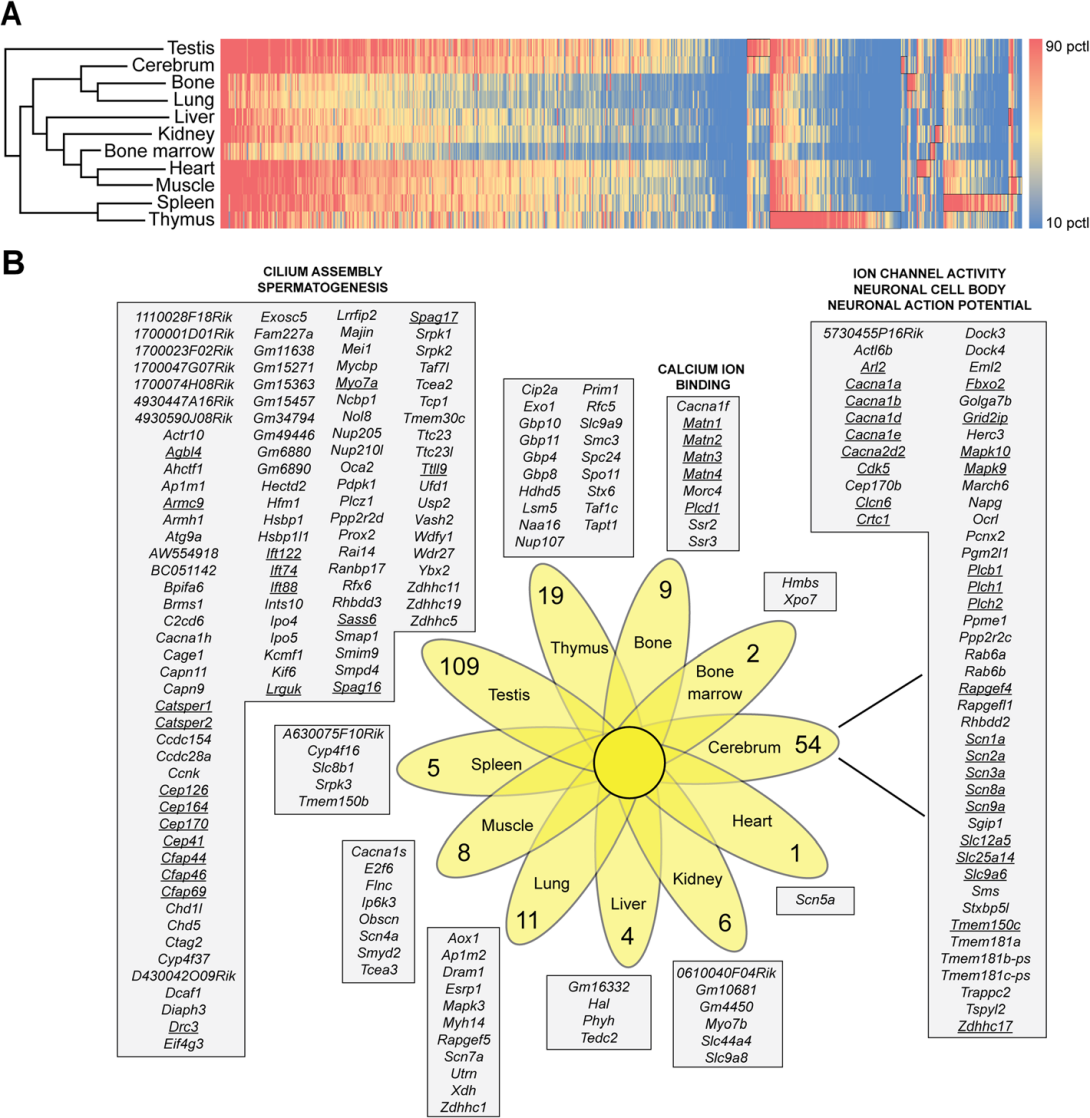
Tissue-enriched MIGs participate in specific biological functions. **a** Hierarchical clustering of mouse tissues based on MIG expression. Heatmap represents TPM values of all 666 MIGs. **b** Venn diagram showing the number of uniquely upregulated (>2FC; *P* < 0.01) MIGs in each tissue. Genes enriched in each tissue are listed, as well as the biological processes that were significantly enriched for. MIGs participating in any of the biological processes are underlined. pctl = percentile

To determine which tissues were most similar in their MIG expression pattern, we performed hierarchical clustering of the mouse tissues based on the expression of all 666 MIGs (Fig. 3a). The analysis revealed that testis was an outlier, followed by thymus and spleen, which were closely paired (Fig. 3a). Similarly, the heart and skeletal muscle were closely related, as well as bone and lung. The heatmap of MIG expression showed several clusters of MIGs that were highly expressed in one tissue compared to the other tissues (Fig. 3a). To identify the MIGs that were specifically enriched in one tissue, and thus form the expression signature of that tissue, we employed a bioinformatics pipeline which we named *SignatureCalc*. Here, we extracted all MIGs that were significantly upregulated (>2FC) in one tissue compared to all other samples to generate the UpSignature (Additional file 1: Figure S1). Similarly, we extracted all MIGs that were significantly downregulated (>2FC) in one tissue compared to all others (DownSignature) (Additional file 1: Figure S1). Using this approach, we found 228 MIGs that were upregulated in one tissue compared to all others (Fig. 3b). Most of these were enriched in the testis (109) and cerebrum (54), but each interrogated tissue showed enriched expression of at least one MIG (Fig. 3b). To determine whether their expression would contribute to a tissue-specific function, we submitted all UpSignature gene lists for functional annotation analysis by DAVID, g:Profiler and Toppgene [14–16]. Only those functions that were identified as significant enrichments by all three tools were reported. We found that the 109 testis-upregulated MIGs significantly enriched for functions including centriole and cilium assembly (Fig. 3b; Additional file 12: Table S4). Moreover, submission of the 54 MIGs in the UpSignature of the cerebrum to functional annotation tools showed several significant enrichments, including voltage-gated ion channel activity and neuronal cell body (Fig. 3b; Additional file 12: Table S4). This is in keeping with the upregulation of the several members of the *Cacna*, *Scn* and *Slc* gene family in the cerebrum compared to the other ten tissues (Fig. 3b). Submission of the remaining UpSignature lists revealed only one more significant enrichment: MIGs enriched in bone are important for calcium ion binding (Fig. 3b; Additional file 12: Table S4). Next, we determined the DownSignature, i.e. MIGs specifically downregulated in one tissue compared to all others, for each sample. Several tissues did not have a DownSignature, or in other words, did not express MIGs at a significantly lower level than all other tissues. However, we did find 23 MIGs expressed at >2FC lower in bone marrow, 20 MIGs in testis, and 108 MIGs in the liver (Additional file 2: Figure S2B). Submission of the MIGs downregulated in the liver enriched for functions including voltage-gated ion channel activity and cilium morphogenesis (Additional file 12: Table S4). In sum, we found tissue-enriched expression of many MIGs, which often encode for proteins important for the function of the tissues in which they are upregulated.

### Shorter minor introns are retained at a higher level in a tissue-specific manner

Besides being regulated at the transcriptional level, MIGs are thought to be regulated at the level of splicing, specifically minor intron splicing. Currently, the model for minor intron splicing postulates that minor introns are generally spliced inefficiently, resulting in retention and introduction of a premature stop codon [17]. This would then either activate the nonsense-mediated decay (NMD) pathway or result in the production of an aberrant protein isoform. However, this model has emerged from analysis performed on few MIGs, such as *P120*, *PROX1*, *SCN4A*, *INSIG2*, *SNRPE* and *E2F2* [8, 17–20]. We wanted to investigate whether this form of regulation extends to all MIGs or whether it is intron-specific, as suggested by recent RNAseq analyses. Notably, RNAseq from zebrafish, maize, our U11 cKO mouse, Roifman syndrome and myelodysplastic syndrome patients has shown that there is a wide range of minor intron retention levels in absence of a fully functioning minor spliceosome [4, 6, 21–23]. These reports highlight that inefficient minor intron splicing as a means to regulate MIG expression might not extend to all MIGs.

**Fig. 4:**
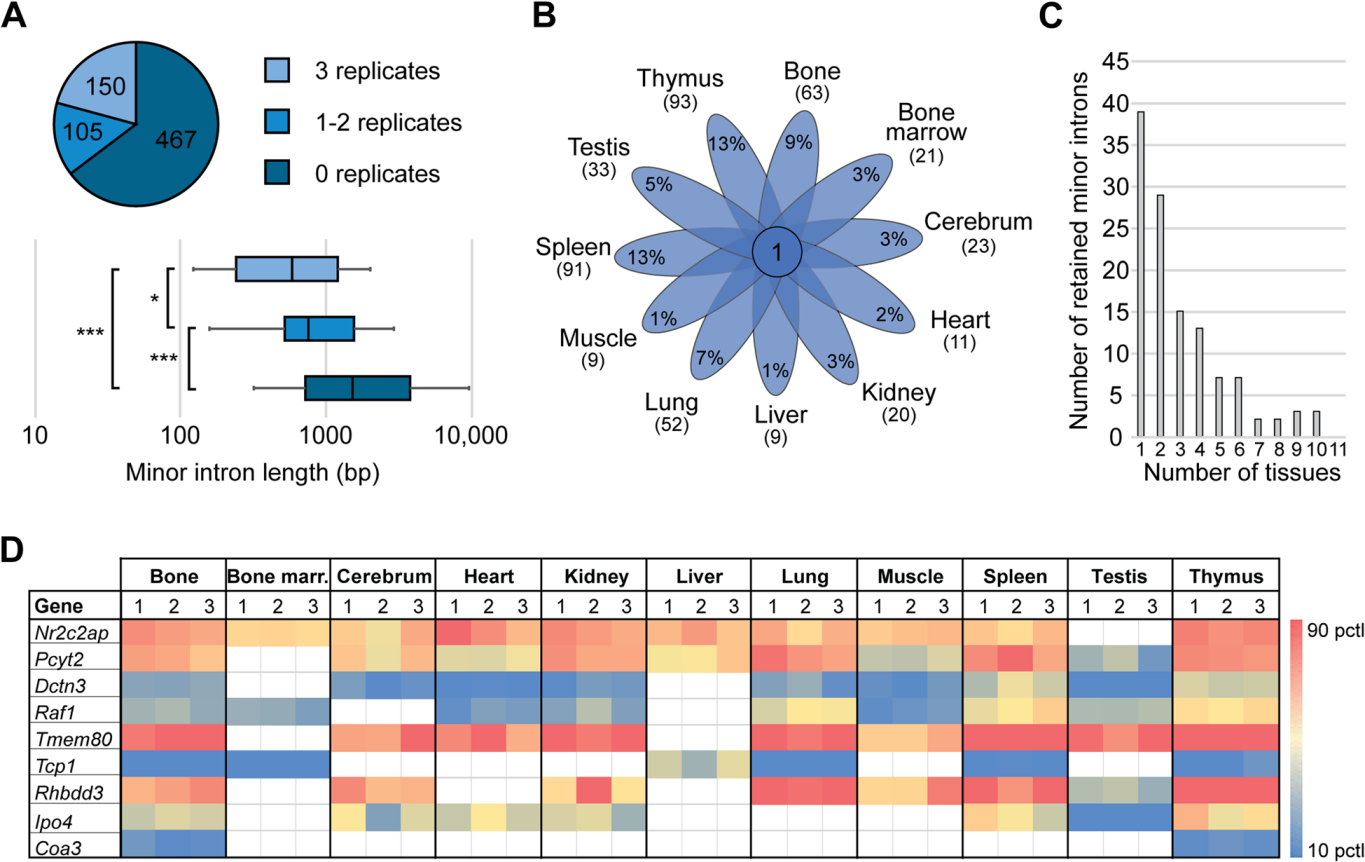
Minor introns are retained in a tissue-dependent manner. **a** Piechart with number of minor introns that show retention in number of replicates of at least one tissue. Boxplots reflect the 5th–95th percentile of minor intron length in each of the three categories. Significance was determined by Kruskal-Wallis rank sum test, followed by post-hoc multiple comparison using Dunn method. *** = *P* < 0.001; * = *P* < 0.05. **b** Venn diagram showing the overlap of retained minor introns across eleven mouse tissues. Only minor introns that passed filtering criteria in all three replicates of a tissue were included. **c** Histogram of the number of tissues in which minor introns were retained. **d** Heatmap of MSI values across eleven mouse tissues for nine minor introns. White cells denote that a minor intron did not pass the filtering criteria for retention in that tissue. See also methods. MSI = mis-splicing index; marr. = marrow; pctl = percentile

To test which MIGs showed minor intron retention at steady-state in WT tissues, and thus could be regulated through inefficient minor intron splicing, we next employed the method described by Madan et al. [22]. Briefly, we quantified the exon-minor intron boundary reads to determine minor intron retention levels, which were reported as a mis-splicing index (MSI) (Additional file 3: Figure S3A) [22]. Using our filtering criteria (Additional file 3: Figure S3A), we found that 150 minor introns were retained in all three replicates of at least one tissue, 105 minor introns were retained in 1 or 2 replicates in at least one tissue, while 467 minor introns did not show any retention at all in any of the eleven tissues (Fig. 4a). Next, we wanted to interrogate whether the retained minor introns contained specific features, making them more susceptible to retention. First, we interrogated the effect of intron length on the level of minor intron retention. Here, we found that the 150 retained minor introns were significantly shorter than the 467 minor introns that did not show any retention (Fig. 4a). Since minor introns were initially identified based on their divergent AT-AC consensus sequences, we next wanted to determine whether retained minor introns contained different splice sites than those minor introns that were not retained. However, analysis of the 5′ splice site (SS), branch point (BP) and 3′ SS sequences revealed similar consensus sequences regardless of the retention status (Additional file 4: Figure S4).

To establish whether minor introns were subject to tissue-specific splicing, resulting in dynamic retention across tissues, we determined the number of retained minor introns in each tissue. To this end, we made conservative calls by limiting ourselves to minor introns that showed evidence of retention in all three replicates. Only one of these 150 minor introns showed retention in all interrogated tissues (Fig. 4b). In fact, most minor introns showed dynamic use of retention across tissues. Specifically, only 9 minor introns were retained in the liver, while there were 93 minor introns retained in the thymus (Fig. 4b). To exclude the possibility that this variability was caused by tissue-specific expression of MIGs, we focused only on those minor introns that were found in MIGs expressed > 1 TPM in all eleven tissues. Of the remaining 120 retained minor introns, none showed retention across all interrogated tissues (Fig. 4c). Moreover, only three minor introns were retained in 10 tissues, and for a large portion of the 120 minor introns retention was only observed in a single tissue (Fig. 4c). Furthermore, we found that the level of retention for the same minor intron varied across different tissues (Fig. 4D; Additional file 13: Table S5). For example, *Pcyt2* showed minor intron retention in ten tissues, but at significantly different levels. Notably, it was retained at the highest MSI levels in kidney, lung, spleen, and thymus, which was significantly higher than the retention levels in heart, skeletal muscle and testis (Fig. 4D; Additional file 13: Table S5). In contrast, *Tcp1*, which showed retention in six tissues, had significantly higher MSI values in the liver compared to its retention levels in bone, bone marrow, lung, spleen and thymus (Fig. 4D; Additional file 13: Table S5). Thus, MIGs are not only differentially expressed across tissues, but their minor intron is also spliced in a tissue-specific manner.

Importantly, splicing of minor introns also occurred in a tissue-dependent manner in humans. Like in mouse, few minor introns showed retention in at least 3 replicates of one tissue (Additional file 5: Figure S5A). In fact, hardly any minor intron retention was detected in the human cerebral cortex, heart, kidney and liver, which were also tissues with low minor intron retention in mouse (Fig. 4B; Additional file 5: Figure S5B). It must be noted however that some of these human tissues had a low sequencing depth, which could contribute to the fact that we detected so few retained introns (Additional file 16: Table S8). Moreover, for most retained minor introns we only detected minor intron retention in one or two tissues, specifically testis and bone marrow (Additional file 5: Figure S5B-C). This is unlike mouse, where spleen and thymus contained the most retained minor introns (Fig. 4b). In addition, the minor introns that are retained in the bone marrow, spleen and testis are mostly distinct between mice and human (Additional file 5: Figure S5D). Thus, while the expression patterns of MIGs seemed conserved between the two species, retention of minor introns did not. However, we did find that shorter introns were also more often retained than longer minor introns in human (Additional file 5: Figure S5A).

### Minor intron splicing can be efficient

While major intron retention used to be considered the consequence of mis-splicing, it is now considered an actively regulated form of alternative splicing (AS) that often serves a physiological function [24]. However, in the case of minor introns, retention is still thought to be the result of inefficient splicing caused by low levels of minor spliceosome snRNPs or slower splicing rates [1, 17]. As such, at steady-state, one might expect the splicing of major introns flanking the minor intron to be more efficient than that of the minor intron. Indeed, this was the finding reported by Patel et al., where they analyzed the rate of major intron versus minor intron retention for three MIGs through qRT-PCR analysis [17]. Since the model was based on a small sample size, we wanted to extend this analysis to all MIGs.

**Fig. 5:**
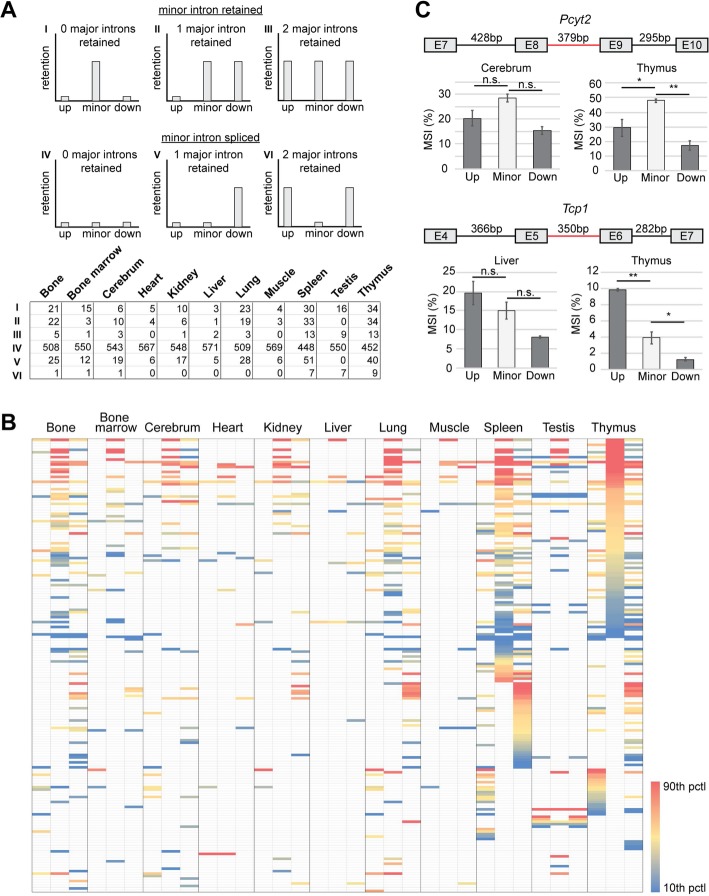
Minor intron splicing can be efficient. **a** Table showing the number of retained minor introns and flanking major introns across eleven mouse tissues. Above are schematics exemplifying the 6 different categories. **b** Heatmap of MSI values for minor introns, upstream major introns, and downstream major introns across eleven mouse tissues. **c** Bargraphs showing retention levels for the minor intron, upstream and downstream major introns of *Pcyt2* and *Tcp1* in different tissues as determined by RNAseq. Data are represented as mean ± SEM. Above is the gene schematic with intron sizes. Minor intron is depicted by the red line. MSI = mis-splicing index; E = exon; pctl = percentile. Significance was determined by one-way ANOVA, followed by post-hoc Tukey test. * = *P* < 0.05; ** = *P* < 0.01; n.s. = not significant

Of the 722 minor introns in the mouse genome, 582 minor introns are flanked by both an upstream and downstream major intron (Additional file 6: Figure S6). The remaining minor introns were either found as the first or last intron in the gene (122), flanked by a major and a minor intron (12), or were the only intron in the gene (6) (Additional file 6: Figure S6). One such MIG that contains a minor intron without any major introns, is *Coa3.* This suggests that splicing and indirectly expression of these MIGs solely relies on the minor spliceosome, yet retention of this single minor intron was tissue-specific (Fig. 4d). Next, we wanted to analyze the MSI values for the upstream and the downstream major intron flanking the minor intron, and perform statistical analysis to compare these levels to that of minor intron retention. Thus, for this analysis we were limited to the 582 minor introns that were flanked by two major introns. We first wanted to establish for how many of the retained minor introns in each tissue there was no retention of the flanking major introns, as this would be consistent with the model of inefficient minor intron splicing. We found that, if a minor intron was retained in a tissue, the flanking major introns were frequently not retained (Fig. 5a; Bin I). However, for most minor introns we did not detect retention, while in several cases the flanking major introns were retained (Fig. 5a; Bin V-VI), which suggests that minor intron splicing is not always inefficient. For example, in thymus, a tissue with the most retained minor introns, we also found nine minor introns that were spliced efficiently, yet in those cases both of the flanking major introns showed retention (Fig. 5a; Bin VI). In addition, we found that even when a minor intron is retained, its levels are not always higher than that of the flanking major introns (Fig. 5b; Additional file 14: Table S6). For instance, *Pcyt2,* which has a minor intron of 379 bp and flanking major introns of similar size, showed significantly elevated minor intron retention compared to that of the flanking major introns in the thymus. However, in the cerebrum, this elevated minor intron retention is not significant (Fig. 5c), which would be in agreement with a model of dynamically regulated minor intron splicing. A more extreme example is *Tcp1,* which contains a minor intron of 350 bp. In the thymus, this minor intron was retained at low levels (Fig. 5c). However, when we compared it to the retention levels of the flanking major introns, we found that the upstream major intron had significantly elevated retention compared to the minor intron (Fig. 5c). Again, this effect is tissue-specific as we did not find similar significant changes in the liver (Fig. 5c). In all, our analysis showed that in vivo, minor intron retention as a consequence of inefficient splicing might not apply to all MIGs.

### Minor introns are alternatively spliced in a tissue-dependent manner

**Fig. 6:**
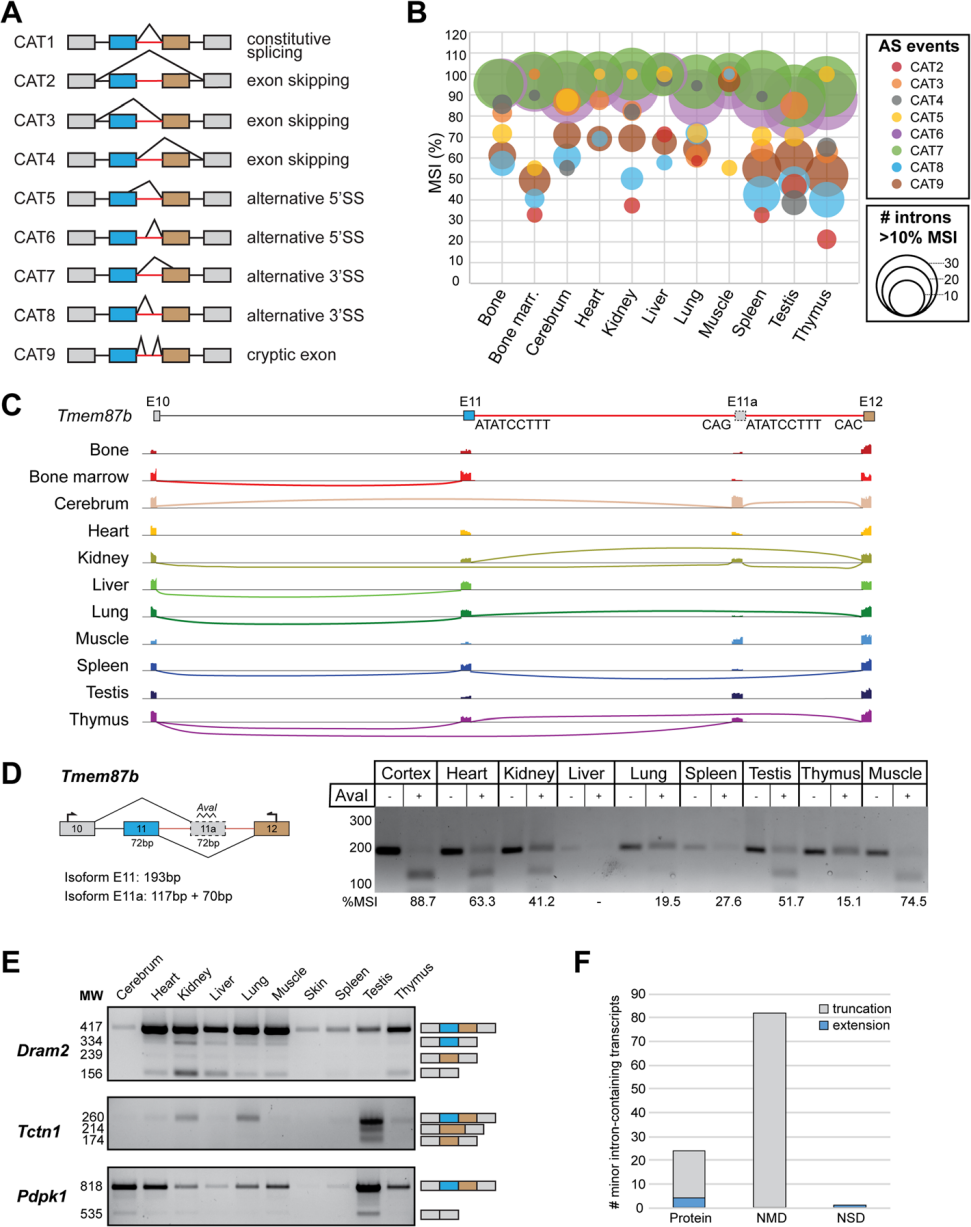
Minor introns are alternatively spliced in a tissue-dependent manner. **a** Schematic of the nine interrogated AS events. Red line denotes the minor intron. **b** Bubbleplot reflecting AS usage across minor introns in eleven mouse tissues. Size of the circle represents the number of introns that passed the filtering criteria, the colour represents the type of AS. See also Methods. **c** Sashimi plot of *Tmem87b* showing tissue-dependent usage of a cryptic exon within the minor intron. Only arcs representing > 10 reads were included. **d** Gel image of RT-PCR products for *Tmem87b* across different tissues. Products were cut with AvaI restriction enzyme to determine tissue-specific isoform usage. Predicted product sizes are shown below the gene schematic. Red line denotes the minor intron. %MSI was calculated using ImageJ by quantifying respective band intensity. **e** Gel images of RT-PCR products for *Dram2, Tctn1* and *Pdpk1* across different tissues. Schematic of identified transcripts on the right. **f** Bargraph representing the number of alternatively spliced transcripts predicted to be translated into protein, or targeted for non-sense mediated decay (NMD) or non-stop mediated decay (NSD). MSI = mis-splicing index

Our discovery that minor intron retention is a dynamically regulated event across tissues, supports the idea that minor intron retention is not just the result of inefficient splicing. Instead, we propose to consider it as a type of alternative splicing, as is now accepted for major intron retention [25]. As such, we next wanted to determine whether other forms of AS events would occur across minor introns in a tissue-specific manner. Previous reports have detected few AS events across minor introns [1], which may be explained by the bioinformatics tools employed for the detection of AS events. For example, a widely used tool to detect AS events is MISO [26–28], which detects AS events that have been annotated previously, thereby missing new AS events [29]. Here we did not want to limit our search to annotated AS events, so we curated all spliced reads that mapped around the minor intron. We then quantified the number of reads that would support constitutive splicing (CAT1, Fig. 6a) or any of the eight different AS events (Fig. 6a). It must be noted that we interrogated each of these events separately. However, because these events are not necessarily mutually exclusive, a single read can support multiple AS events. We then determined the level of each AS event for each minor intron in each tissue, represented as an MSI value (Additional file 3: Figure S3B). Using this approach, we found that most of the eight AS categories were utilized in all eleven tissues (Fig. 6b).

The most prominent form of AS that was observed around minor introns involved cryptic splicing. Specifically, CAT6 and CAT7 (Fig. 6b; Additional file 15: Table S7), which represent an alternative 5′SS and 3′SS (Fig. 6a), respectively, were used at a high level in all tissues. In contrast, exon skipping (CAT2–4) was detected at lower levels in most tissues and was often employed in a dynamic manner across tissues (Fig. 6b). For instance, five MIGs showed evidence of exon skipping in the testis, while not a single exon skipping event around minor introns was observed in the bone, cerebrum, heart and skeletal muscle (Fig. 6b). Finally, the introduction of a cryptic exon within the minor intron (CAT9) was observed in several MIGs, albeit at lower levels (Fig. 6b). Again, the dynamic usage of this form of AS was illustrated by the fact that it was utilized by 15 MIGs in the thymus, yet by only 4 MIGs in the skeletal muscle (Fig. 6b). Overall, we detected very few AS events across minor introns in the heart, liver and skeletal muscle, which is similar to our observation of low levels of minor intron retention in these tissues (Fig. 4b; Fig. 6b). In contrast, many AS events were observed in the spleen and thymus, the tissues that also possessed the most retained minor introns (Fig. 4b; Fig. 6b). Finally, despite having detected only few retained minor introns in testis, we did observe the most AS events in this tissue compared to other tissues (Fig. 4b; Fig. 6b). In all, these results show that AS across minor introns is utilized in a tissue-dependent manner and that these AS events are more common than previously thought [1]. Consistent with these observations, is our finding that ~ 8% of the annotated isoforms of MIGs in the Ensembl database employ AS events across the minor introns (Additional file 7: Figure S7A-B) [30]. Moreover, we identified a positive correlation between alternative splicing and the length of the minor intron, such that alternatively spliced minor introns were significantly longer than those that were not alternatively spliced (Additional file 7: Figure S7C).

### Identification of alternatively spliced MIGs

**Fig. 7:**
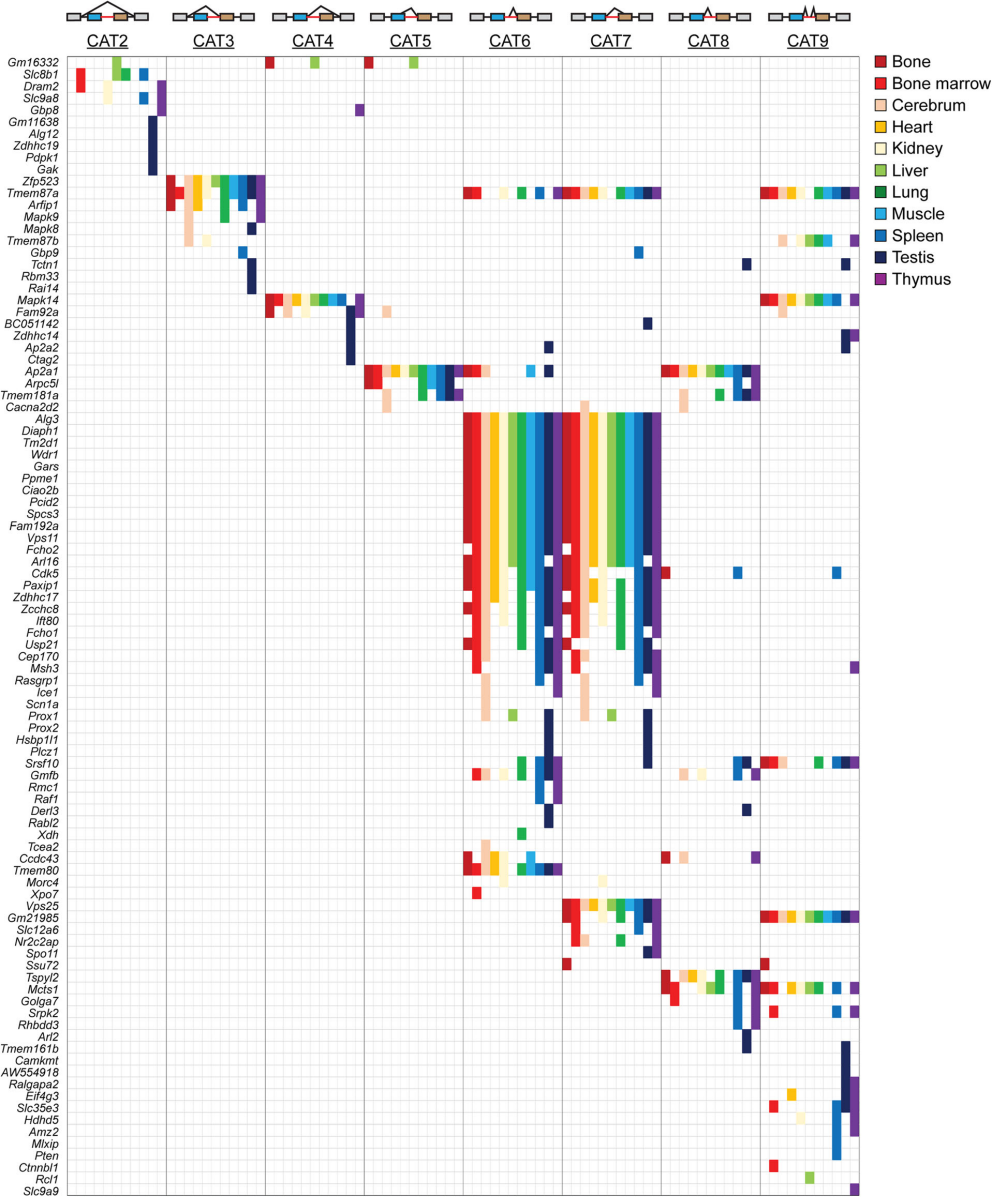
Multiple AS events around the minor intron are employed within the same MIG. Table showing the minor introns that are alternatively spliced. Colours indicate the tissue that a specific AS event was detected in for a minor intron

Since multiple AS events can be used in conjunction in a single MIG, we next took a MIG-centric approach. This analysis revealed that utilization of a cryptic 5′SS (CAT6) was often coincident with a cryptic 3′SS (CAT7) (Fig. 7). There were 12 minor introns where CAT6 was not used along with CAT7, while nine minor introns used a cryptic 3′SS (CAT7) but not CAT6 (Fig. 7). Similarly, a cryptic 5′SS resulting in a truncation of the upstream exon (CAT5) was often utilized in combination with a cryptic 3′SS resulting in extension of the downstream exon (CAT8) (Fig. 7). Types of AS that were generally employed as a single event in a MIG included all forms of exon skipping. Specifically, skipping of both the upstream and downstream exon (CAT2) was the sole AS event found in eight MIGs (Fig. 7). Moreover, we found ten MIGs that expressed an isoform in which the upstream exon was skipped (CAT3). These included mitogen-activated protein kinases, and *Tmem87a* and *Tmem87b* (Fig. 7)*.* Especially the skipping event in the mitogen-activated protein kinases was tissue-specific, as it was highly upregulated in the cerebrum compared to other tissues (Fig. 6b; Fig. 7). While the AS around the minor introns of mitogen-activated protein kinases had previously been described [31], many other AS events were undescribed so far. For instance, the AS event around the minor intron of *Tmem87b*, which is located between exons 11 and 12, was a novel event. We observed skipping of exon 11 (CAT3) in the cortex and the kidney (Fig. 7). In addition, we detected the presence of a cryptic exon 11a within the minor intron of *Tmem87b* (Fig. 7). Sashimi plots revealed that these events were not resulting in separate isoforms but were connected, such that when exon 11 is skipped, the cryptic exon 11a is utilized (Fig. 6c). Moreover, we did not observe any spliced reads spanning exon 11 and exon 11a, which suggested that these two exons are mutually exclusive (Fig. 6c). We performed RT-PCR to confirm that in the cortex, the most robustly expressed transcript of *Tmem87b* is the one that skips exon 11 and utilizes exon 11a (Fig. 6d). However, both exon 11 and 11a are 72 bp, which with the primer pairs we designed resulted in PCR-products of identical size (Fig. 6d). Therefore, we used the unique AvaI restriction site found in exon 11a to determine the presence of isoforms containing exon 11a but not exon 11, which is resistant to this digest. These results support the RNAseq analysis in that the predominant isoform expressed in the cortex is the one with exon 11a (Fig. 6d). Importantly, the minor spliceosome is also required to splice intron 11a, as supported by the U12-type 5′SS, despite the fact that the annotated minor intron is skipped. In addition, we validated several other AS events around minor introns. For example, in the testis, we detected skipping of exons flanking the minor intron in *Pdpk1* and *Tctn1* by RNAseq (Fig. 7). RT-PCR analysis confirmed the robust presence of these isoforms in the testis, as well as low levels in several other tissues, such as the cerebrum (Fig. 6e). Similarly, we had detected skipping of both exons flanking the minor intron of *Dram2* (CAT2) in bone marrow, kidney and thymus by RNAseq (Fig. 7). Analysis by RT-PCR confirmed the presence of the isoform in these tissues, but also showed low expression of isoforms generated by different alternative splicing events around the minor intron of *Dram2* (Fig. 6e). Thus, besides confirming RNAseq results, we were also able to detect several additional isoforms expressed at low levels. In other words, our RNAseq analysis was conservative in the identification of AS events around minor introns, in order to minimize false positives. Overall, we identified several novel AS events around minor introns that were often used in conjunction and resulted in the expression of tissue-specific isoforms.

To test whether these isoforms could also result in the production of tissue-specific proteins, we performed bioinformatics analysis to predict the fate of alternatively spliced transcripts. RNA transcripts are under tight quality control, such that aberrant splicing can result in activation of several degradation pathways. For instance, intron retention can result in nuclear retention of the transcript and subsequent degradation by the exosome [32]. Alternatively, aberrant transcripts are exported to the cytoplasm where they can be degraded via pathways such as NMD and/or non-stop decay (NSD) [33]. It has been predicted that transcripts containing a premature stop codon > 50 nt upstream of an exon-exon junction are subjected to NMD, whereas transcripts lacking a stop codon before the polyA-signal would be subject to NSD [34, 35]. Thus, we used these criteria to predict whether the alternatively spliced isoforms would be translated into protein, or degraded via NMD or NSD. We found that the majority of alternative splicing events would introduce a premature stop codon, resulting in truncation of the open reading frame (ORF) and most likely degradation via NMD (Fig. 6f). In contrast, only one transcript was predicted to result in NSD (Fig. 6f). Lastly, ~ 22% of the AS events are predicted to result in transcripts that could be translated into proteins. Of these, the vast majority would result in truncation of the protein, compared to the canonical isoform (Fig. 6f). Only four AS events were predicted to result in an extension of the protein, potentially resulting in novel protein domains (Fig. 6f).

Finally, we also wanted to determine the prevalence of AS around minor introns in humans. Again, we found that cryptic splicing of minor introns (CAT6 and CAT7) was used at the highest levels in all tissues (Additional file 8: Figure S8A). In addition, we observed dynamic usage of AS across minor introns in the investigated tissues (Additional file 8: Figure S8A; Additional file 15: Table S7). Specifically, we detected exon skipping (CAT2) in many MIGs in the bone marrow and testis, but not in other tissues (Additional file 8: Figure S8A; Additional file 15: Table S7). Moreover, skipping of the downstream exon (CAT4) was used for more minor introns in the bone marrow than in any other tissue (Additional file 8: Figure S8A; Additional file 15: Table S7). In addition, we found that the usage of a cryptic exon (CAT9) was used for a varying number of minor introns across the tissues, suggesting that AS across minor introns is also a regulated event in humans (Additional file 8: Figure S8A). Overall, the most minor introns were alternatively spliced in the bone marrow and the testis (Additional file 8: Figure S8A). Comparison of alternatively spliced MIGs between mouse and human revealed that 46 MIGs were alternatively spliced in both mouse and human, whereas 36 MIGs were alternatively spliced in at least one tissue in mouse, and 81 MIGs were only alternatively spliced in human (Additional file 8: Figure S8B). Thus, even though AS across minor introns also occurs in humans, the specific AS events for each individual minor intron do not seem to be conserved and its prevalence varies between the species. However, we cannot exclude the possibility that this difference stems from a difference in age between the two species.

## Discussion

The field of minor intron splicing is gaining prominence with discoveries of diseases linked to mutations in genes encoding the components of the minor spliceosome [4, 5, 36, 37]. In order to understand the mechanism of disease pathogenesis in these syndromes, one must have a detailed overview of the MIGs that could have been altered by the inhibition of the minor spliceosome. In this regard, the U12DB has served a valuable role in providing an easily accessible list of minor introns and MIGs. However, the U12DB has not been updated since 2007, while several new genome assemblies have been released since [10, 30]. Here, we present a new list of minor introns, which was generated using PWMs as described by Sheth et al. [11]. The new list of MIGs captures 99 and 97% of all MIGs reported in the U12DB for the mouse and human genome, respectively and thus is not expected to fundamentally change the results of previous studies. However, the identification of new MIGs, which is likely the result of new genome assemblies that refined the exon/intron boundary coordinates, does expand the list of target genes that may underlie phenotypes created by minor spliceosome-related diseases. As such, it would be interesting to see whether any of the newly identified minor introns are affected in these diseases.

Given that we identified 666 MIGs in the mouse genome, it is not surprising that MIG expression is tissue-specific. However, when MIG expression was used to cluster tissues, the relationships between these tissues were different than when total gene expression was used to perform the same analysis (Fig. 3a; Additional file 2: Figure S2A). This tissue-specific MIG expression might be one of the contributing factors to the tissue-specificity observed in the diseases caused by mutation in the various minor spliceosome components [2, 3, 22, 36–38]. Since cerebrum and testis expressed the most MIGs at the highest levels (Fig. 2a-d), one may predict these tissues to be most susceptible to loss of minor intron splicing. Indeed, one of the cardinal symptoms of MOPD1 and Roifman syndrome, which are caused by mutation in the U4atac snRNA, is microcephaly [2–4]. Defects in testis development have not been reported in MOPD1 patients, but mutation in the RRM of *Zrsr1*, a retrotransposed copy of *Zrsr2*, has been shown to result in impaired spermatogenesis [28]. Specifically, *Zrsr1-*mutant testis contain seminiferous tubules arrested at the spermatocyte stage, as well as sperm with abnormal tail development, which suggests defects in their cilium assembly [28]. Interestingly, MIGs upregulated in the testes significantly enriched for cilium assembly (Fig. 3b), which underscores the importance of understanding tissue-specific expression of MIGs. Indeed, tissues with low MIG expression such as liver and heart have not been reported in patients and/or animal models with inactivated minor spliceosome. Thus, it appears that liver and heart manage without a functioning minor spliceosome and aberrant MIG expression. Together, these findings suggest that the differential transcriptional regulation of MIGs might contribute to tissue-specific phenotypes in minor spliceosome-related disease.

While we observed tissue-specific transcriptional regulation of MIG expression, the more widely investigated mechanism for the regulation of MIG expression is through retention of their minor intron(s) [17]. The inherent complexity of coordinating both the major and the minor spliceosome to splice introns co-transcriptionally, combined with the inefficient splicing of minor introns is thought to result in the retention of minor introns [17]. In keeping with this model, our RNAseq analysis of WT tissues showed 150 minor introns that were retained in at least one tissue (Fig. 4a). However, we only observed retention for one minor intron in all tissues (Fig. 4b), which raises the possibility that minor intron retention is not a stochastic output of inefficient splicing, but rather an actively regulated process. For example, we found that the minor intron in *Pyct2* is retained at a high level in cerebrum, but retention was not detected in bone marrow (Fig. 4d). In all, these data support a model of active minor intron splicing regulation.

We favor such a model, which would mean that splicing and/or retention of minor introns can behave like the splicing of major introns. This model is in contrast to the previous model of stochastic or inefficient minor intron splicing resulting in prevalent retention of minor introns. The latter ideas were based on comparison of minor intron retention levels to that of the flanking major introns. Briefly, Patel et al., employed this comparison to test the hypothesis that minor introns are excised more slowly than the upstream and/or the downstream major introns [17]. They showed by qPCR analysis that for three human MIGs (*SNRPE, E2F2, INSIG1*), minor introns were retained at a higher level than the flanking major introns [17]. Afterwards, the Frilander group used RNAseq of Hep-2 cells to show that, on average, minor introns are indeed retained at higher levels than their flanking major introns, thereby supporting this finding [27]. However, the variability of retention of individual minor introns was not addressed in this study; even though a wide spread of minor intron retention was observed [27]. While we detected few minor introns that could support the idea of inefficient minor intron splicing, our model is supported by the observation that retention of several minor introns is lower than their upstream and/or downstream major introns (Fig. 5). Thus, the model of inefficient minor intron splicing does not apply to all MIGs and the decision to retain an intron, be it major or minor, is a highly regulated process in MIGs.

The apparent differences in our findings and the subsequent models could be explained by the choice of MIGs that were interrogated. We found that retention of shorter minor introns was detected at a higher level than the longer introns (Fig. 4a). This finding is in line with a recent report by Pieczynski et al., who showed that splicing efficiency of the minor intron in the Arabidopsis gene *CBP20* became progressively more efficient by increasing the length of the intron [39]. Together, these findings suggest that efficiency of minor intron splicing, as reported by intron retention, is inversely proportional to the intron length, which has been reported previously for major introns [40]. The elevated levels of retention in shorter minor introns is especially relevant in light of the knowledge that most of the MIGs used to study minor intron retention contained short introns, as they are amenable to RT-PCR analysis. Indeed, in one of the first papers on minor intron splicing where a P120 splicing substrate was used, the authors mentioned that: “This particular minor class intron was selected because of its small size” [18]. Since then, *P120* has served as a golden standard for studying the efficiency of minor intron splicing. Thus, the confluence of a naturally occurring preference for retention of shorter minor introns and the choice of minor introns that were studied for technical reasons might have led to the minor intron retention as a model for regulating all MIGs.

Given our observation that splicing of minor introns is highly regulated, it stands to reason that it is also tissue-specific. Indeed, we found dynamic levels of minor intron retention across different mouse tissues (Fig. 4d; Additional file 13: Table S5). For example, we found that minor introns were most often retained in the mouse thymus and spleen, and in human bone marrow (Fig. 4b; Additional file 5: Figure S5C). This is in agreement with previous reports which discovered that immune cells and neuronal cells generally have the highest intron retention levels [40]. Since lymphoid organs already possess high levels of minor intron retention normally, this suggests that these tissues would experience highly elevated minor intron retention in disease states. Support for this idea is evidenced by the fact that the lymphatic system is commonly affected in patients with minor spliceosome-related diseases such as myelodysplastic syndrome (MDS) and Roifman syndrome [4, 22]. In addition, systemic inhibition of the minor spliceosome in mouse through loss of the protein Rnpc3, also resulted in immunological defects [41]. Specifically, Doggett et al. showed that systemic postnatal ablation of *Rnpc3* resulted in leukopenia and a severely reduced thymus lacking medullary or cortical regions [41]. Together, actively regulated, tissue-dependent minor intron retention, could contribute to the tissue-specific phenotypes observed in diseases with systemic inhibition of the minor spliceosome.

If minor intron retention is an actively regulated process, one might predict that it would respond to stress or other environmental cues. Interestingly, Mauger et al. showed that neurons contain large amounts of stable intron-retained transcripts in their nucleus that are not rapidly degraded by nuclear decay mechanisms [42]. Upon activation of the neural network by incubation with bicuculline, these introns were spliced out and the transcripts were loaded onto ribosomes, thus allowing for a rapid increase in protein production. Several activation-dependent splicing events of introns were validated by RT-PCR, including intron 19 of the gene *Ahctf1* [42]*.* Since this intron is a minor intron, it supports the model of actively regulated minor intron retention. Moreover, the Dreyfuss group has shown that stress can enhance minor intron splicing. By using anisomycin to activate the stress-induced protein kinase *Mapk14,* U6atac snRNA levels were increased, and splicing of several minor introns found in MIGs involved in stress physiology was enhanced [26]. Based on these findings, they proposed a model where minor introns can act as molecular switches through retention and alternative splicing [26]. However, the extent to which AS across minor introns occurs remained unclear [1].

Here we have shown that for a subset of MIGs there are extensive tissue-specific AS events (Fig. 6). For example, the skipping events observed for *Tmem87b* (Fig. 6c-d) bring to light the coordinated action of both the major and the minor spliceosome to use the major 5′ SS of intron 10 in conjunction with the 3′SS of an exon found embedded within the annotated minor intron 11a. For this to occur, the immediate major 3′ SS that is available in intron 10 was not used, which suggests an active regulation of AS by the minor spliceosome or an alternative splicing factor. Indeed, *Mapk9*, which has a similar gene arrangement as *Tmem87b*, with a minor intron between exons 7 and 8, also shows mutually exclusive usage of exon 7 and 7a. The isoform that skips the upstream exon, but splices into the cryptic exon has been shown to be enriched in the cortex and regulated by the cortex-specific AS factor Nova2 (Fig. 7) [43]. Although *Tmem87b* and *Mapk9* both employ mutually exclusive exons around the minor intron, and both show exclusive expression of one of the isoforms in the cortex (Fig. 6C; Fig. 7), *Tmem87b* was not found to be a target of Nova2 by CLIPseq [43]. This suggests that there are multiple AS factors that may interact with the minor and major spliceosome in a tissue-specific manner, to regulate AS of MIGs. Moreover, we found that the spleen, testis and thymus contained the most alternative spliced MIGs. This is in contrast with the AS of major introns, which is most frequent in the central nervous system [44]. Thus, the regulation of AS across the minor intron is independent of the frequency of AS across major introns in the same gene. In summary, our investigation provides a new avenue to explore tissue-specific MIG expression through transcription, minor intron retention and other forms of alternative splicing.

## Conclusion

Here we report an updated list of MIGs that is available to the research community at https://midb.pnb.uconn.edu. This database is currently restricted to mouse and human, but will be extended to other species in the future. Investigation into these minor introns showed that their splicing can be efficient compared to that of the flanking major introns, suggesting that retention of minor introns might be considered a form of alternative splicing. This retention and alternative splicing is in fact related to the length of the minor intron, such that shorter introns are more often retained while longer introns are more often alternatively spliced. Importantly, we discovered that the transcription, splicing and alternative splicing of MIGs is highly dynamic across tissues. This discovery has the potential of explaining why diseases linked to inactivating mutations in minor spliceosome components do not result in a systemic defect, but instead show tissue-specific symptoms. Moreover, the expression analysis that we present here can also be used to understand disease symptoms associated with mutations in the MIGs themselves. Finally, our report opens a new line of thought that brings minor intron splicing and the regulation of MIGs in the context of systems biology.

## Methods

### Bioinformatics analysis

#### Classification of minor introns

To identify additional minor introns, we scored the splice sites of all annotated introns against position-weighted matrices of major and minor introns, as reported previously by Sheth et al. [11]. Briefly, all annotated intron in the mouse and human genome were curated from Ensembl (v.95). We then isolated the consensus sequences at the 5′ and 3′ splice sites for all introns. Here, we used a stretch of 12 nucleotides for the 5′SS (− 3 to + 9), and 14 nucleotides for the 3′SS (− 13 to + 1). The branch point sequence (BPS) of minor introns has been shown to be closely positioned to the 3′SS, with an optimal distance of 11–13 nucleotides [45]. As such, we curated a sequence up to 40 nucleotides upstream of the 3′SS (− 40 to − 1) and then isolated 29 windows of 12 nucleotides in length, thus resulting in 29 putative BPS.

Position probability matrices (PPMs) for the 5′SS and 3′SS of minor introns (AT-AC and GT-AG type) and major introns (GT-AG and GC-AG type) were downloaded from SpliceRack [11]. Moreover, two different PPMs for the BPS of minor introns were curated, one with the adenosine at the 9th, and one with the adenosine at the 10th position. Each element *M* in these matrices was then transformed to create a PWM using the following formula: ${M}_{k,j}={\log}_2\frac{M_{k,j}}{0.25}$. Any 0 values in the PPMs were replaced by a pseudo count of 0.0001.

The curated splice sites of all introns were then scored against the PWMs for minor introns and major introns by adding the scores of each individual element ($m=\sum \limits_{j=1}^n{M}_{k,j}\Big)$. The obtained scores for each intron were then rescaled to a scale from 0 to 100 using the following formula for negative scores: $x=50\ast \frac{m-{m}_{min}}{-{m}_{min}}$ and for positive scores: $x=50+50\ast \frac{m}{m_{max}}$. This way negative values were transformed to be from 0 to 50, and positive values were transformed to be on a scale from 50 to 100.

For the identification of minor introns we employed similar criteria as mentioned by Sheth et al. [11]. As such, an intron was classified as a minor intron if it met any of the following 4 criteria:

1. The score for the minor intron AT-AC 5′SS PWM was over 50, and at least 10 points higher than the score for any other 5′SS PWM.
2. The score for the minor intron GT-AG 5′SS PWM was over 50, and at least 25 points higher than the score for the major intron GT-AG 5′SS PWM, as well as 10 points higher than the score of the other 5′SS PWMs
3. The score for the minor intron GT-AG 5′SS PWM was over 50, and at least 10 points higher than the score for any other 5′SS PWM. Moreover, the score for the minor intron BPS PWM was over 65.
4. The sum of the scores for the minor 5′SS, BPS and 3′SS PWM was over 150.

We then isolated those minor introns that would need to be spliced out in order to generate the canonical transcript. For this we used the Ensembl definition of the canonical transcript. In other words, the CCDS translation, or otherwise Ensembl/Havana translation, with the longest coding sequence (CDS) would be considered the canonical translation. If these were not present, then any transcript with the longest CDS would be considered the canonical isoform. If there was no CDS, then the longest non-protein coding transcript was assigned as the canonical transcript.

#### Data accession

Data analyzed in this study was downloaded from the DDBJ and EMBL database. They can be accessed using the following accession numbers: Mouse WT tissues (DRA005768; DDBJ database) [12]; Human tissues (E-MTAB-2836; EMBL database) [13]. Information on number of replicates and sequencing depth can be found in Additional file 16: Table S8.

#### Gene expression analysis

Reads from human tissue samples were aligned to the hg38 genome (UCSC genome browser) using Hisat2 as described previously [6, 46]. Reads obtained from mouse tissue samples were aligned to the mm10 genome (UCSC genome browser). Gene expression was then determined by IsoEM2, as described previously [6, 47]. Hierarchical clustering was performed using average linking with Genesis [48].

#### SignatureCalc

Differential gene expression analysis was conducted using IsoDE2, as described previously [6, 47]. Genes were considered differentially expressed if the fold-change was > 2 with *P* < 0.01. Using this method, 55 pairwise comparisons were performed between all mouse tissues. Afterwards, the intersect was taken of all the upregulated genes. Genes that were binned as upregulated in all pairwise comparisons, were classified as being part of that tissue’s UpSignature. Conversely, genes that were binned as downregulated in one tissue compared to all other tissues were classified as being part of that tissue’s DownSignature (Additional file 1: Figure S1).

#### Minor intron retention analysis

Coordinates of flanking major introns from the canonical transcript were determined using Ensembl (v.95) and utilized for the splicing efficiency analysis in Fig. 4. To determine minor and major intron retention in mouse and human tissues, we employed the strategy previously described by Madan et al. [22]. Here, retention is described as the mis-splicing index (MSI), which was calculated for each intron using BEDTools [49]. Only introns that passed the filtering criteria (> 4 exon-intron boundary reads, ≥1 read aligning to both the 5′ and 3′ splice site, > 95% intron coverage) in all replicates of a condition were considered to be retained (Additional file 3: Figure S3A).

#### Alternative splicing analysis

To detect AS across minor introns, we extracted all uniquely mapped spliced reads spanning the minor intron. Reads mapping with only one nucleotide on either side of the junction were excluded. The remaining spliced reads were then binned into one of 9 splicing categories (Fig. 6a) using BEDTools [49]. First, spliced reads supporting the canonical exon-exon junction were binned as CAT1. This was followed by binning the spliced reads supporting one of the three exon skipping categories. Reads spanning the minor intron and flanking exons but not mapping to either, were classified as CAT2. Reads skipping the upstream exon but aligning to the minor intron and/or downstream exon were classified as CAT3. Similarly, reads skipping the downstream exon, but mapping to the minor intron and/or upstream exon, were binned as CAT4. Then, BEDTools was employed to extract all reads supporting cryptic splice site usage. To this end, only reads mapping to one exon and supporting a cryptic splice site > 2 nt from the canonical splice site at the other exon were included. Afterwards, the specific coordinates for these novel exon-exon junctions were utilized as bait to extract additional spliced reads that mapped to this cryptic splice site but not the opposite exon. Finally, spliced reads mapping within the minor intron and also mapping to either of the flanking exons were binned as CAT9, cryptic exon usage. We then calculated the relative AS usage for each AS event for each minor intron as the mis-splicing index (MSI). This was determined as the percentage of reads supporting an individual AS event (Additional file 3: Figure S3B). To reduce false positives, we only considered minor introns to be alternatively spliced if the average MSI of all replicates for a given condition was > 10% (Additional file 3: Figure S3B). Moreover, a threshold for the average number of reads supporting an AS event was employed by normalizing to the sequencing depth. Specifically, only AS events with > 1 read per 3 million uniquely mapped reads were included (Additional file 3: Figure S3B).

#### Functional enrichment analysis

UpSignature lists for each tissue were submitted for functional enrichment analysis to DAVID, ToppGene and g:Profiler [14–16]. Significant GO-Terms (Benjamini-Hochberg adjusted *P*-value< 0.05 for DAVID; FDR-corrected *P*-value< 0.05 for ToppGene; g:SCS threshold< 0.05 for g:Profiler) were isolated. Only GO-Terms that were significant according to all three functional annotation tools are reported in Fig. 3 and Additional file 10: Table S2. Similar analysis was performed for the DownSignature lists.

#### Open Reading frame (ORF) analysis

For all alternatively spliced MIGs, we determined the new exons of the canonical transcript depending on the specific AS event that was observed. If multiple AS events were observed around a minor intron, these were treated as separate events. Moreover, if the read filter was passed for an AS event, as result of multiple coordinates being employed, but no single coordinate passed the read filter separately, the event was excluded from this analysis. An example would be the use of a cryptic 5′SS in a minor intron (CAT6) which was supported by 20 reads, however this was the sum of reads all supporting different coordinates being employed as a cryptic 5′ SS. The transcripts of the remaining protein-coding genes were then translated in silico and the open reading frame was predicted. The location of the new stop codon was compared with the annotated stop codon to determine whether the AS event had resulted in a truncation or extension of the ORF. If the new stop codon was found > 50 nt upstream of an exon-exon junction, the alternatively spliced isoform was predicted to be degraded by NMD. If no stop codon was identified, then the transcript was predicted to be degraded by NSD. In all other cases, the transcript was predicted to give rise to a protein.

#### Annotated isoform analysis

To determine whether AS events across minor introns had been annotated, we downloaded the exon coordinates of all MIG transcripts from Ensembl (v.95). Exon coordinates of each transcript were then compared to the minor intron coordinates and transcripts were binned into one of four categories: 1) transcripts that supported constitutive splicing of the minor intron, 2) transcripts that were truncated before the minor intron coordinates, 3) transcripts with their first/last exon within the minor intron coordinates, and 4) transcripts with AS across the minor intron.

#### Statistical analyses

To determine whether the median length of retained vs. spliced minor introns were statistically different, we performed a Kruskal-Wallis rank sum test, followed by post-hoc Dunn test. A one-way ANOVA, followed by post-hoc Tukey test was employed for determining dynamic levels of retention for individual minor introns across tissues. To determine whether minor introns were retained at significantly higher levels than their flanking major introns, we also performed one-way ANOVA, followed by post-hoc Tukey test. Finally, to determine whether there was a significant difference in the median length of alternatively spliced vs. canonically spliced minor introns, we performed a Mann Whitney U-test.

### Transcardial perfusion

Eight week old C57/Bl6J male mice (*N = 3*) were obtained from The Jackson Laboratory and anesthetized using isoflurane, and perfused using 0.125 M NaCl as described previously [50]. All mouse procedures were performed according to protocols that conformed to NIH guidelines for the use and care of animals in research, which were approved by the University of Connecticut Institutional Animal Care and Use Committee (IACUC).

### RNA extraction and cDNA preparation

Ten tissues were dissected from 8-week-old C57/Bl6J perfused male mice (*N = 3*). Cortex, heart, kidney, liver, lung, and testis were homogenized in 1 mL TRIzol (#15596018); skin, spleen, thymus and tibialis anterior (skeletal muscle) were homogenized in 500 µL TRIzol. RNA was extracted using the DirectZOL RNA Miniprep kit (#R2062, Zymo Research), per the manufacturer’s instructions. 1μg of total RNA was then used for cDNA synthesis, as described previously [51].

### RT-PCR

25 ng of cDNA prepared from total RNA was used for reverse-transcriptase PCR (RT-PCR) analyses. For AS analysis in pan tissue, cDNA from three replicates was pooled. MSI values were determined by quantification of band intensity by ImageJ.

## Additional files

Additional file 1: **Figure S1.** *SignatureCalc* pipeline. Schematic describing the pipeline to determine the UpSignature and DownSignature for bone. This pipeline was then run to determine these signatures for the other ten mouse tissues. NonDE = non-differentially expressed. (TIF 599 kb)

Additional file 2: **Figure S2.** Functional enrichment of DownSignatures. (**A**) Dendogram showing the hierarchical clustering of mouse tissues based on overall gene expression. (**B**) Venn diagram showing the number of uniquely downregulated (>2FC; *P* < 0.01) MIGs in each tissue. (TIF 3875 kb)

Additional file 3: **Figure S3**. Bioinformatics strategy for detection of retention and AS of minor introns. (**A**) Schematic describing the pipeline to detect minor intron retention, including filtering criteria. (**B**) Schematic describing the pipeline to detect novel AS events across minor introns, including filtering criteria. Adopted from Madan et al., 2015 [22]. See also Methods. MSI = mis-splicing index; SS = splice site; M = million. (TIF 561 kb)

Additional file 4: **Figure S4.** Minor intron retention is not dependent on consensus sequence. Frequency plots of the annotated 5′SS, BPS and 3′SS of minor introns that are retained in 3 (top), 1 or 2 (middle), or 0 (bottom) replicates of at least one tissue. (TIF 5672 kb)

Additional file 5: **Figure S5.** Minor intron retention is tissue-specific in human tissues. (**A**) Piechart with number of minor introns that show retention in number of replicates of at least one tissue. Boxplots reflect the 5th–95th percentile of minor intron length in each of the three categories. Significance was determined by Kruskal-Wallis rank sum test, followed by post-hoc multiple comparison using Dunn method. ** = *P* < 0.001; n.s. = not significant. (**B**) Histogram of the number of tissues in which minor introns were retained. (**C**) Venn diagram showing the overlap of retained minor introns across eight human tissues. Only minor introns that passed filtering criteria in at least three replicates of a tissue were included. **(D)** Stacked bargraph showing the number of retained minor introns in each tissue in mouse (blue), human (red), or both (grey). (TIF 10985 kb)

Additional file 6: **Figure S6.** Location of minor introns within canonical transcript. Piechart showing the percentage of minor introns that have flanking major introns. (TIF 2247 kb)

Additional file 7: **Figure S7.** Alternative splicing across minor introns is annotated in the Ensembl database. (**A**) Bar graph showing the number of annotated isoforms in the Ensembl database which resulted from AS across the minor intron. (**B**) Venn diagram showing the combined usage of AS events in annotated isoforms that are alternatively spliced across the minor introns. **(C)** Boxplots reflect the 5th–95th percentile of minor intron length in minor introns that are and are not alternatively spliced. Significance was determined by Mann Whitney U test. ** = *P* < 0.01. Transcr. = transcription. (TIF 11148 kb)

Additional file 8: **Figure S8.** Alternative splicing across minor introns in human tissues is dynamic. (**A**) Bubbleplot reflecting AS usage across minor introns in 8 human tissues. Size of the circle represents the number of introns that passed the filtering criteria, the colour represents the type of AS. (**B**) Venn diagram revealing the overlap of MIGs that are alternatively spliced in at least one tissue between mouse and human. MSI = mis-splicing index. (TIF 6629 kb)

Additional file 9: **Table S1.** Minor intron coordinates. Contains the coordinates of the identified minor introns in mouse and human, as reported on MIDB. (XLSX 85 kb)

Additional file 10: **Table S2.** MIG orthologs. Contains information on MIG orthologs between mouse and human. (XLSX 81 kb)

Additional file 11: **Table S3.** MIG expression. Contains the expression of MIGs across mouse and human tissues in TPM. (XLSX 208 kb)

Additional file 12: **Table S4.** Functional annotation. Contains the functional annotations enriched for by the UpSignature and DownSignature of mouse tissues. (XLSX 15 kb)

Additional file 13: **Table S5.** Minor intron retention. Contains the MSI values of retained minor introns across tissues. (XLSX 23 kb)

Additional file 14: **Table S6.** Flanking major intron retention. Contains the MSI values of retained upstream and downstream major introns flanking the minor intron. (XLSX 53 kb)

Additional file 15: **Table S7**. Alternative splicing around minor introns. Contains the MSI values of alternatively spliced minor introns in mouse and human. (XLSX 58 kb)

Additional file 16: **Table S8.** RNAseq sample information. Contains information on sequencing depth and mapping percentage of all RNAseq samples used. (XLSX 13 kb)

## Data Availability

The datasets analysed during the current study are available in the DDBJ repository #DRA005768, and EMBL repository #E-MTAB-2836 at http://trace.ddbj.nig.ac.jp/DRASearch/submission?acc=DRA005768 and https://www.ebi.ac.uk/arrayexpress/experiments/E-MTAB-2836/.

## Abbreviations

AS: Alternative splicing
BPS: Branch point sequence
MDS: Myelodysplastic syndrome
MIDB: Minor Intron DataBase
MIG: Minor intron-containing gene
MOPD1: Microcephalic osteodysplastic primordial dwarfism type 1
MSI: Mis-splicing index
NMD: Nonsense-mediated decay
NSD: Non-stop decay
ORF: Open reading frame
PWM: Position-weight matrix
SS: Splice site
