# Supplementary figures and images for: Minor intron splicing revisited: identification of new minor intron-containing genes and tissue-dependent retention and alternative splicing of minor introns
Source: BMC Genomics. 2019 Aug 30;20:686. doi: 10.1186/s12864-019-6046-x (PMC6717393; doi:10.1186/s12864-019-6046-x)

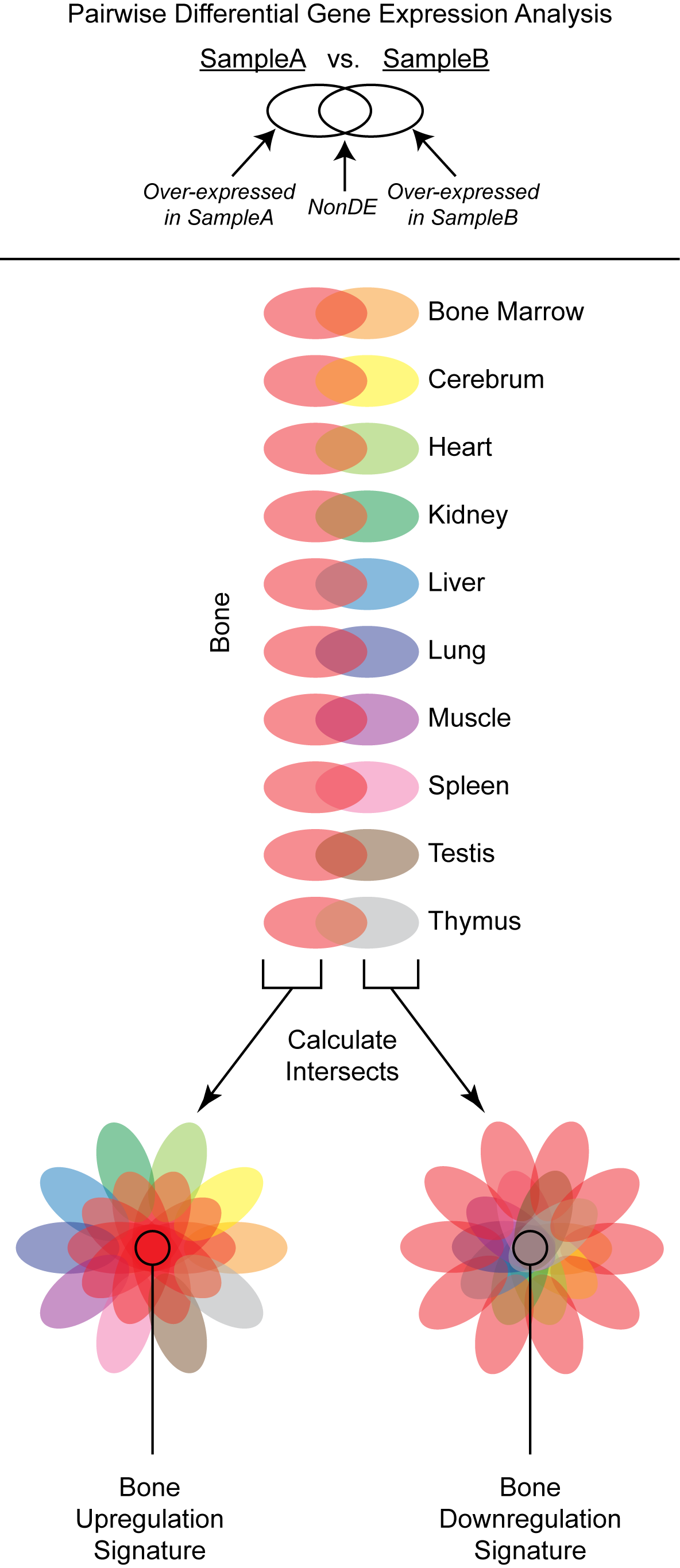

Supplement: Supplementary file 1 — Figure S1. SignatureCalc pipeline. Schematic describing the pipeline to determine the UpSignature and DownSignature for bone. This pipeline was then run to determine these signatures for the other ten mouse tissues. NonDE = non-differentially expressed. (TIF 599 kb) [file 12864_2019_6046_MOESM1_ESM.tif]

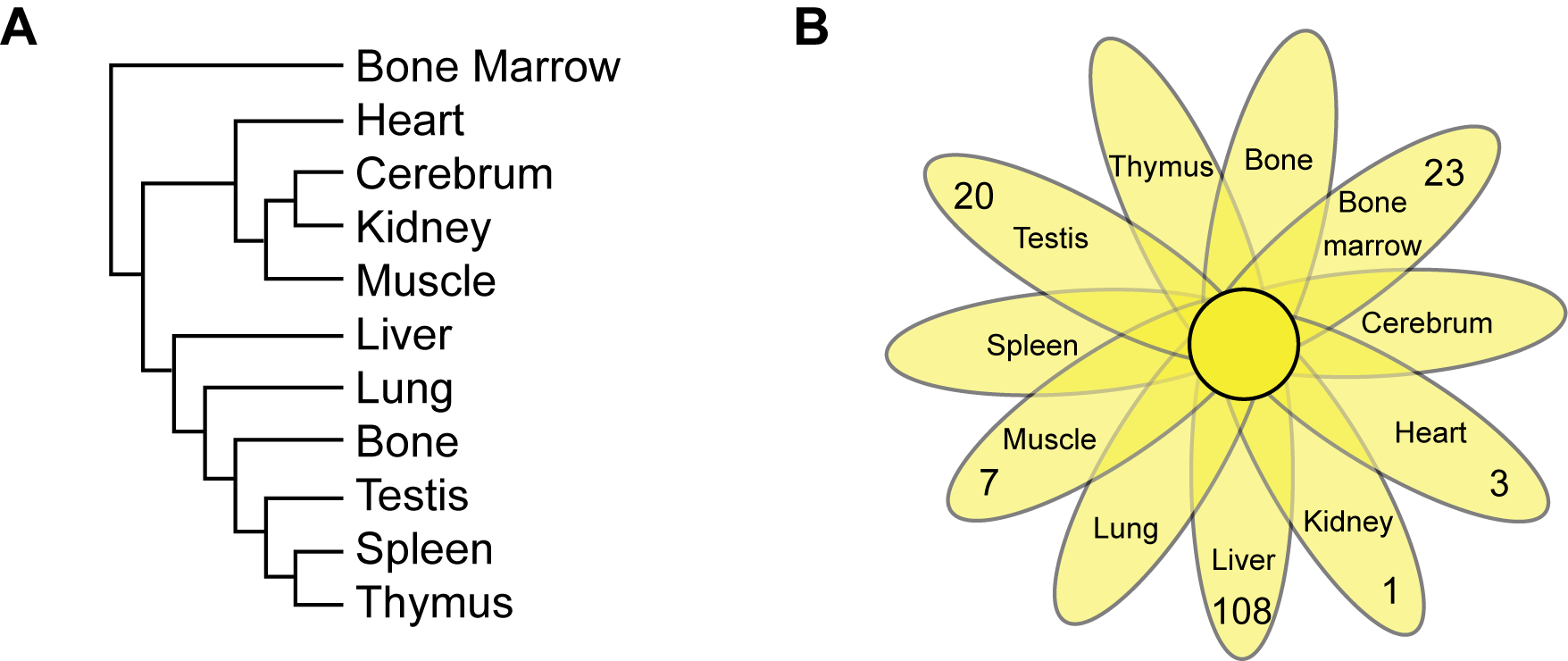

Supplement: Supplementary file 2 — Figure S2. Functional enrichment of DownSignatures. (A) Dendogram showing the hierarchical clustering of mouse tissues based on overall gene expression. (B) Venn diagram showing the number of uniquely downregulated (>2FC; P < 0.01) MIGs in each tissue. (TIF 3875 kb) [file 12864_2019_6046_MOESM2_ESM.tif]

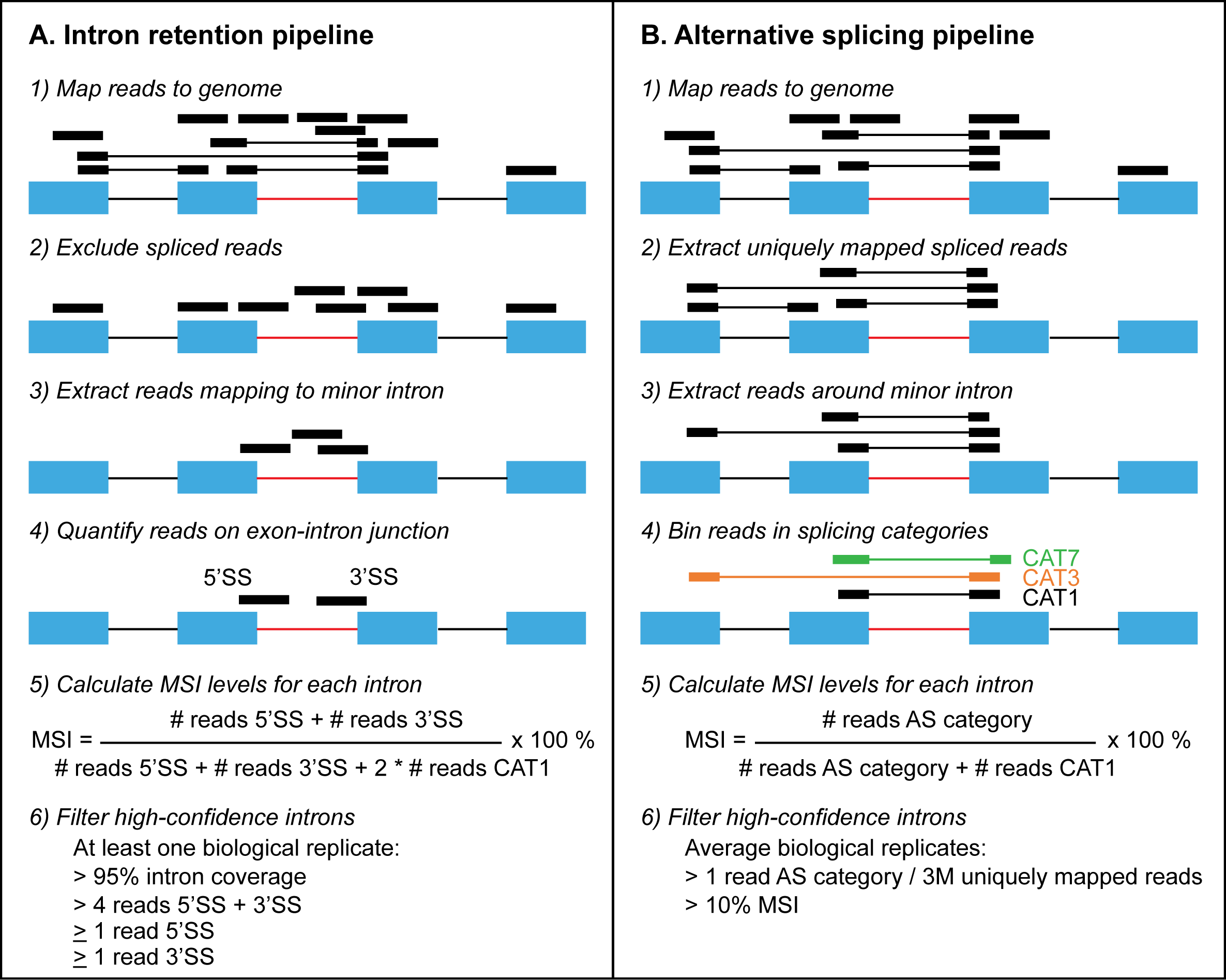

Supplement: Supplementary file 3 — Figure S3. Bioinformatics strategy for detection of retention and AS of minor introns. (A) Schematic describing the pipeline to detect minor intron retention, including filtering criteria. (B) Schematic describing the pipeline to detect novel AS events across minor introns, including filtering criteria. Adopted from Madan et al., 2015 [22]. See also Methods. MSI = mis-splicing index; SS = splice site; M = million. (TIF 561 kb) [file 12864_2019_6046_MOESM3_ESM.tif]

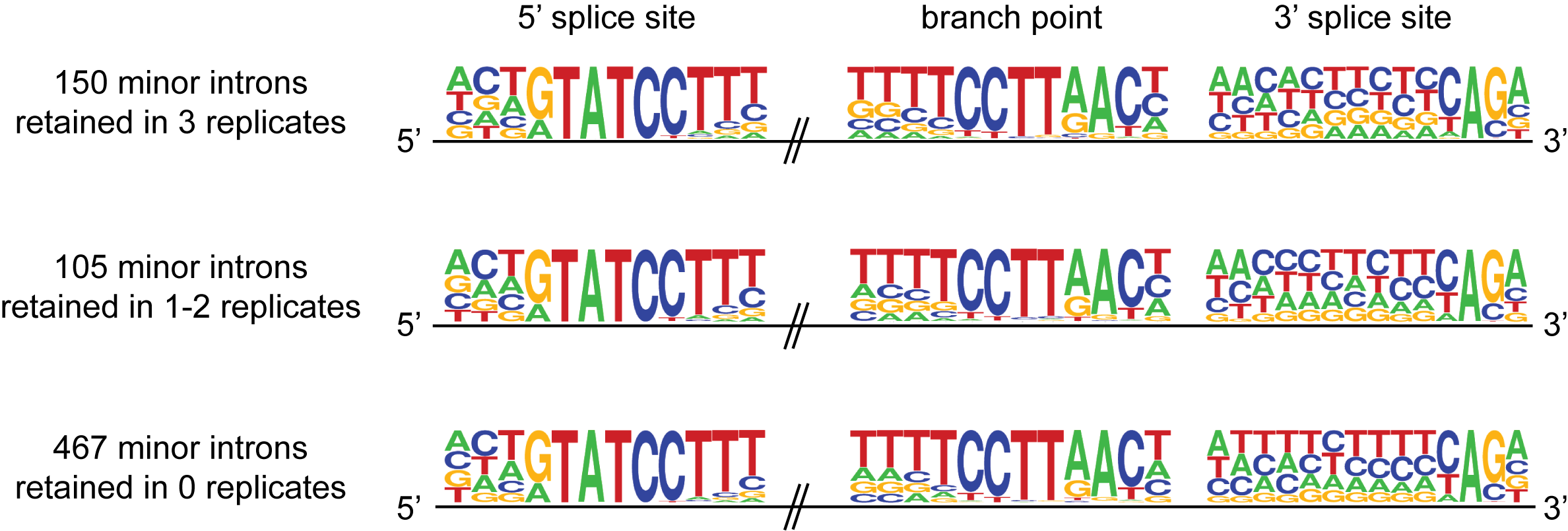

Supplement: Supplementary file 4 — Figure S4. Minor intron retention is not dependent on consensus sequence. Frequency plots of the annotated 5′SS, BPS and 3′SS of minor introns that are retained in 3 (top), 1 or 2 (middle), or 0 (bottom) replicates of at least one tissue. (TIF 5672 kb) [file 12864_2019_6046_MOESM4_ESM.tif]

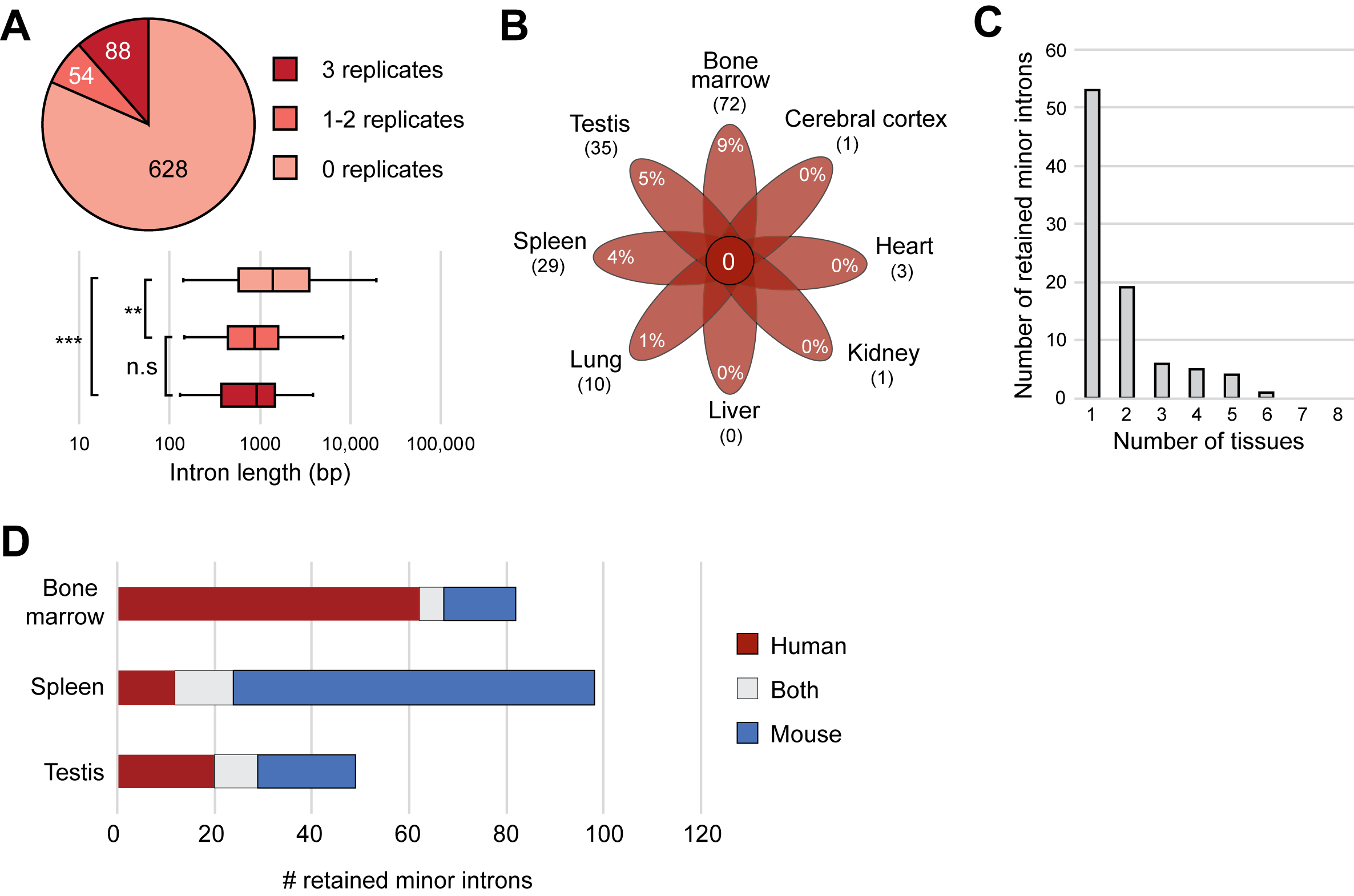

Supplement: Supplementary file 5 — Figure S5. Minor intron retention is tissue-specific in human tissues. (A) Piechart with number of minor introns that show retention in number of replicates of at least one tissue. Boxplots reflect the 5th–95th percentile of minor intron length in each of the three categories. Significance was determined by Kruskal-Wallis rank sum test, followed by post-hoc multiple comparison using Dunn method. ** = P < 0.001; n.s. = not significant. (B) Histogram of the number of tissues in which minor introns were retained. (C) Venn diagram showing the overlap of retained minor introns across eight human tissues. Only minor introns that passed filtering criteria in at least three replicates of a tissue were included. (D) Stacked bargraph showing the number of retained minor introns in each tissue in mouse (blue), human (red), or both (grey). (TIF 10985 kb) [file 12864_2019_6046_MOESM5_ESM.tif]

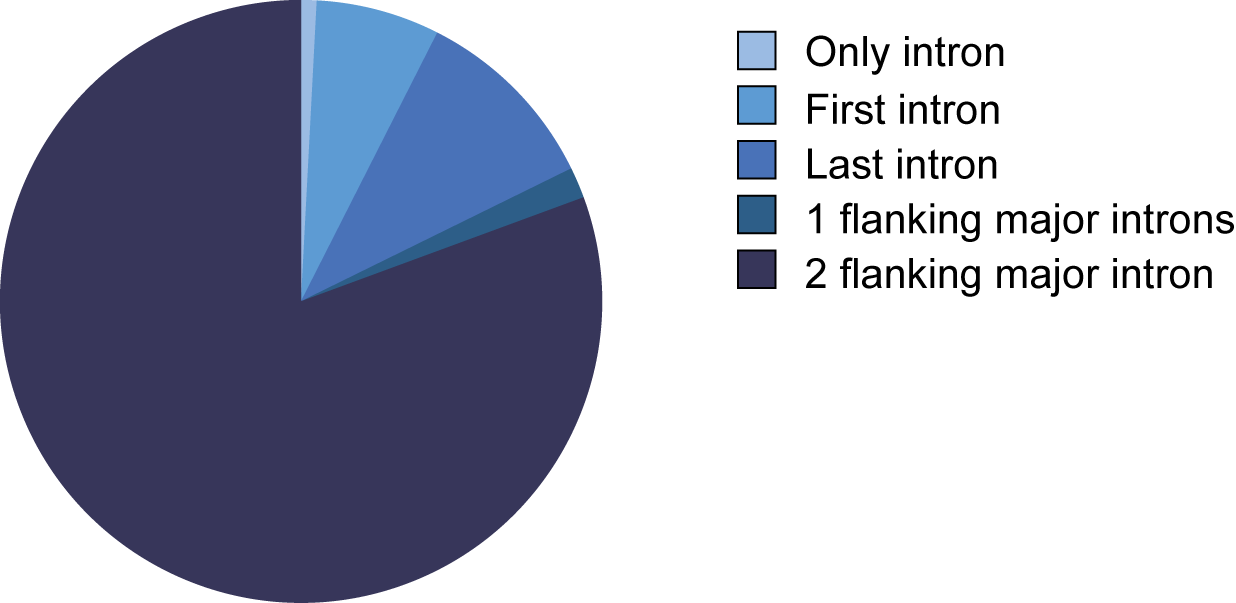

Supplement: Supplementary file 6 — Figure S6. Location of minor introns within canonical transcript. Piechart showing the percentage of minor introns that have flanking major introns. (TIF 2247 kb) [file 12864_2019_6046_MOESM6_ESM.tif]

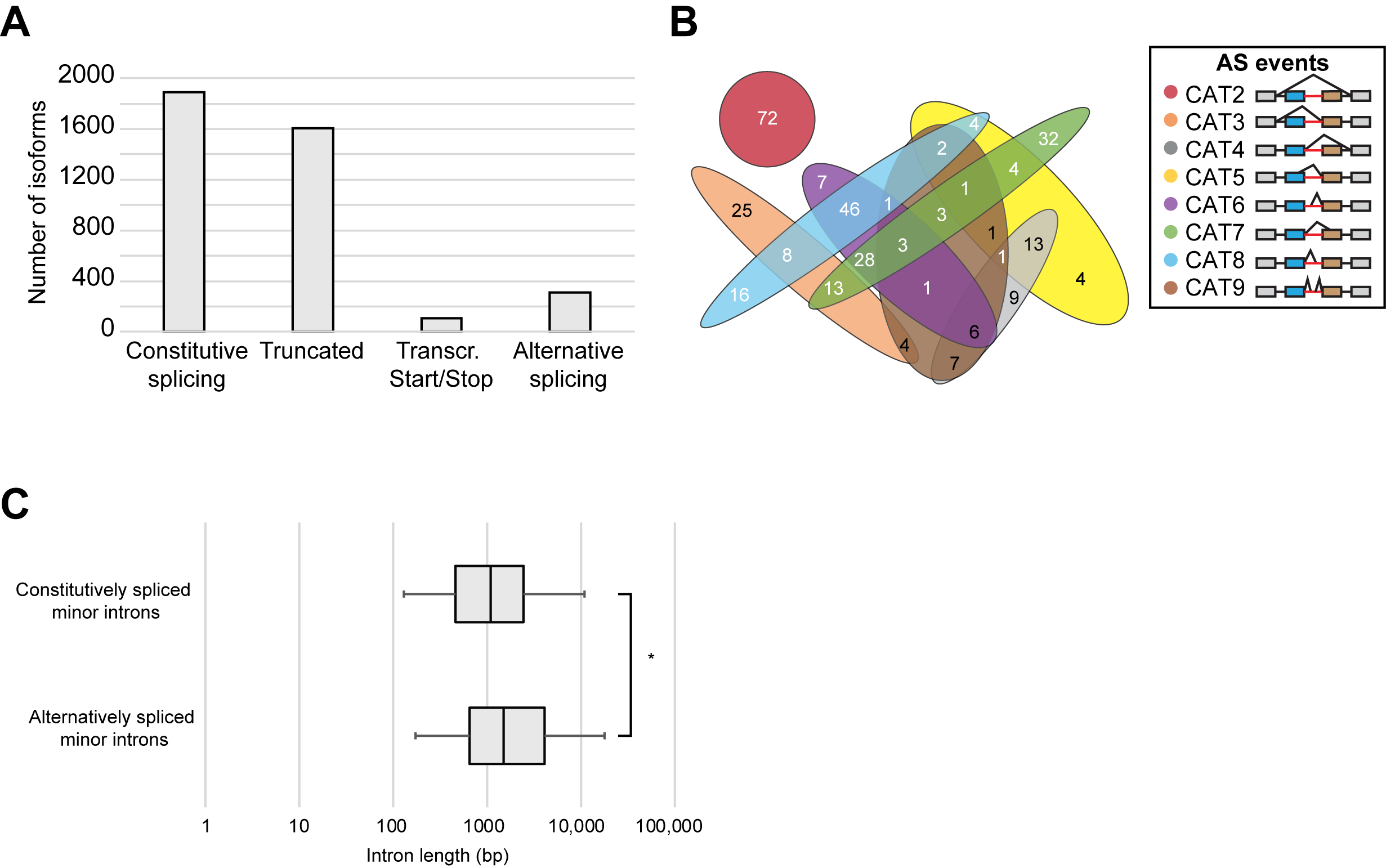

Supplement: Supplementary file 7 — Figure S7. Alternative splicing across minor introns is annotated in the Ensembl database. (A) Bar graph showing the number of annotated isoforms in the Ensembl database which resulted from AS across the minor intron. (B) Venn diagram showing the combined usage of AS events in annotated isoforms that are alternatively spliced across the minor introns. (C) Boxplots reflect the 5th–95th percentile of minor intron length in minor introns that are and are not alternatively spliced. Significance was determined by Mann Whitney U test. ** = P < 0.01. Transcr. = transcription. (TIF 11148 kb) [file 12864_2019_6046_MOESM7_ESM.tif]

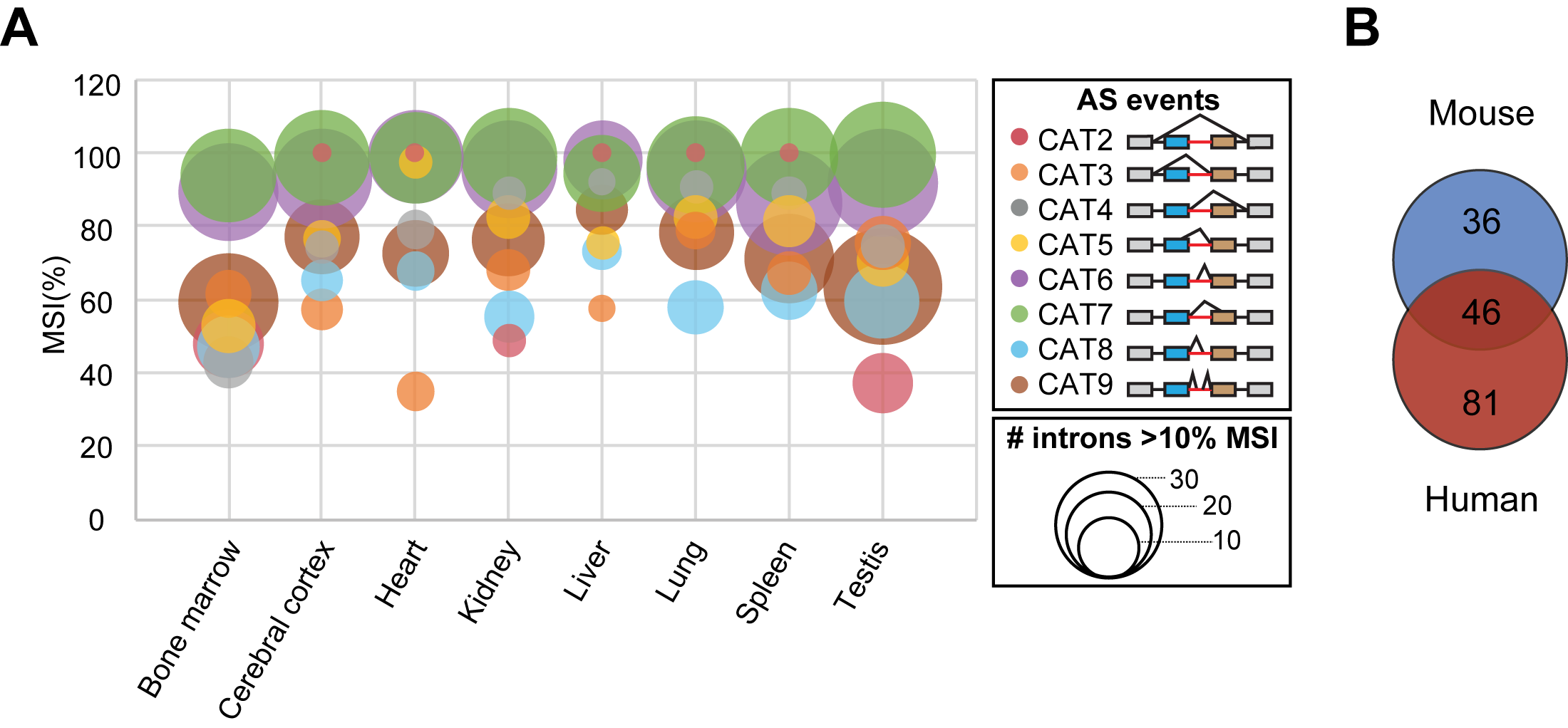

Supplement: Supplementary file 8 — Figure S8. Alternative splicing across minor introns in human tissues is dynamic. (A) Bubbleplot reflecting AS usage across minor introns in 8 human tissues. Size of the circle represents the number of introns that passed the filtering criteria, the colour represents the type of AS. (B) Venn diagram revealing the overlap of MIGs that are alternatively spliced in at least one tissue between mouse and human. MSI = mis-splicing index. (TIF 6629 kb) [file 12864_2019_6046_MOESM8_ESM.tif]
